# Mitigating drought stress in wheat plants (*Triticum Aestivum* L.) through grain priming in aqueous extract of *spirulina platensis*

**DOI:** 10.1186/s12870-024-04905-z

**Published:** 2024-04-02

**Authors:** Mustafa Elnajar, Heshmat Aldesuquy, Mohamed Abdelmoteleb, Eladl Eltanahy

**Affiliations:** 1 Botany Department, Faculty of Science, Mansoura University, Mansoura, 35516 Egypt; 2 Algae Biotechnology Lab, Faculty of Science, Mansoura University, Mansoura, 35516 Egypt

**Keywords:** Drought, *Triticum aestivum*, *S. platensis*, Photosynthetic machinery, Grain yield

## Abstract

**Background:**

The study focuses on the global challenge of drought stress, which significantly impedes wheat production, a cornerstone of global food security. Drought stress disrupts cellular and physiological processes in wheat, leading to substantial yield losses, especially in arid and semi-arid regions. The research investigates the use of *Spirulina platensis* aqueous extract (SPAE) as a biostimulant to enhance the drought resistance of two Egyptian wheat cultivars, Sakha 95 (drought-tolerant) and Shandawel 1 (drought-sensitive). Each cultivar’s grains were divided into four treatments: Cont, DS, SPAE-Cont, and SPAE + DS. Cont and DS grains were presoaked in distilled water for 18 h while SPAE-Cont and SPAE + DS were presoaked in 10% SPAE, and then all treatments were cultivated for 96 days in a semi-field experiment. During the heading stage (45 days: 66 days), two drought treatments, DS and SPAE + DS, were not irrigated. In contrast, the Cont and SPAE-Cont treatments were irrigated during the entire experiment period. At the end of the heading stage, agronomy, pigment fractions, gas exchange, and carbohydrate content parameters of the flag leaf were assessed. Also, at the harvest stage, yield attributes and biochemical aspects of yielded grains (total carbohydrates and proteins) were evaluated.

**Results:**

The study demonstrated that SPAE treatments significantly enhanced the growth vigor, photosynthetic rate, and yield components of both wheat cultivars under standard and drought conditions. Specifically, SPAE treatments increased photosynthetic rate by up to 53.4%, number of spikes by 76.5%, and economic yield by 190% for the control and 153% for the drought-stressed cultivars pre-soaked in SPAE. Leaf agronomy, pigment fractions, gas exchange parameters, and carbohydrate content were positively influenced by SPAE treatments, suggesting their effectiveness in mitigating drought adverse effects, and improving wheat crop performance.

**Conclusion:**

The application of *S. platensis* aqueous extract appears to ameliorate the adverse effects of drought stress on wheat, enhancing the growth vigor, metabolism, and productivity of the cultivars studied. This indicates the potential of SPAE as an eco-friendly biostimulant for improving crop resilience, nutrition, and yield under various environmental challenges, thus contributing to global food security.

**Supplementary Information:**

The online version contains supplementary material available at 10.1186/s12870-024-04905-z.

## Introduction

Wheat (*Triticum aestivum* L.) is a cornerstone of global food security, supplying over one-fifth of total caloric and protein consumption worldwide [1]. Beyond its caloric value, wheat is rich in essential nutrients such as carbohydrates, proteins, vitamins, and minerals [2]. Additionally, grains harbour phytochemicals that synergize with those in fruits and vegetables, promoting a balanced diet [3]. However, the sustainability of wheat production is increasingly threatened by abiotic stressors like drought, which is escalating in frequency and intensity due to climate change and human activities [4], while water drought has led to substantial yield losses, especially in arid and semi-arid regions [5]. By 2025, water scarcity is projected to afflict 1.8 billion people and stress 65% of the global population; consequently, the drought suppresses plant growth at various stages and disrupts cellular and physiological processes, ultimately diminishing grain yield [6].

Photosynthesis, a complex process primarily occurring in chloroplasts, relies heavily on pigments like chlorophylls and carotenoids [7]. These pigments, varying in concentration based on species and environmental conditions, are essential for plants’ adaptation to drought stress. Studies have shown that drought can significantly affect these pigments in wheat, offering reductions in chlorophyll content under water deficit [8–10]. Furthermore, optimal wheat leaf growth is vital in photosynthesis and biomass accumulation. However, it is vulnerable to environmental stresses, particularly drought, which impedes leaf growth through reduced cell expansion and turgor pressure. This results in smaller, less durable leaves and lower leaf biomass due to decreased fresh and dry leaf mass and water content [11]. Moreover, drought conditions significantly affect leaf gas exchange and stomatal behavior, which is critical for photosynthesis and crop yield. Stomatal closure, a drought response, conserves water at the expense of reduced photosynthesis [12]. It also alters carbohydrate metabolism, crucial for plant growth and grain development [13]. Consequently, drought stress significantly reduces wheat yield by affecting key growth stages and crop attributes [14].

Wheat has developed various strategies to cope with water scarcity, including morphological, physiological, and biochemical changes [15]. These adaptations include alterations in root architecture to enhance water uptake [16], stomatal regulation to minimize water loss [17] while optimizing carbon dioxide uptake for photosynthesis [18], and the accumulation of osmoprotectants (such as proline and soluble sugars) that help maintain cell turgor and protect cellular components from drought-induced oxidative stress [19]. Furthermore, wheat plants enhance their antioxidant defense systems under drought conditions to scavenge reactive oxygen species, mitigating oxidative damage and maintaining cellular integrity [20].

Grain priming is a pre-sowing treatment that involves hydrating grains to initiate the pre-germinative metabolism without allowing radicle emergence [21]. This process enhances the physiological state of the grains, leading to improved germination rates, faster seedling growth, and enhanced stress tolerance in plants [22]. In the context of drought stress, grain priming with biostimulants, such as plant extracts or microbial inoculants, can prime wheat grains to better withstand water scarcity by modulating physiological and biochemical responses, enhancing antioxidant activity, and improving water uptake efficiency [23]. Biostimulants, especially those sourced from algal origins, reinforce plant robustness against abiotic challenges such as salinity [24] and drought [25] by *Nostoc muscorum* and *Chlorella vulgaris* extracts [26, 27] while *Spirulina platensis* act as plant biostimulants due to its rich nutrient profile and stress-mitigating properties [28, 29].

Based on existing literature, several mechanisms have been proposed to explain how *S. platensis* mitigates drought-induced physiological declines. These include its antioxidant activity, which neutralizes reactive oxygen species and thus prevents oxidative damage at both the membrane and protein levels [30]. Osmotic adjustment is another mechanism; *S. platensis* aqueous extract (SPAE) treatment increases the accumulation of compatible solutes like sugars, maintaining cell turgor and osmotic balance [31]. Furthermore, antioxidants in SPAE protect the photosynthetic machinery, thereby maintaining photosynthetic efficiency even under drought conditions [32]. Other contributing factors are phytohormone stimulation, which promotes root and shoot (flag leaf) development, and stomatal regulation, which balances water loss and CO_2_ uptake [33]. This study aims to assess the impact of pre-soaking wheat grains in SPAE on the growth and physiological parameters of two wheat cultivars, Shandawel 1 (drought-sensitive) and Sakha 95 (drought-tolerant), under both normal and drought conditions at both heading and harvest levels. Unlike previous investigations that have primarily examined foliar applications or soil amendments with *S. platensis* against salinity, this research uniquely explores the efficacy of grain priming with 10% SPAE for 18 h as a novel strategy for improving wheat performance under drought stress with two cultivars (Shandawel 1 and Sakha 95) provides critical insights into the differential responses of wheat genotypes to biostimulant treatment under water-limited conditions as a new risk in agriculture around the world.

## Materials and methods

### Plant material

Pure strains of two wheat cultivars, the most tolerant (Sakha 95) and most sensitive ones (Shandawel 1), were obtained from the Egyptian Agricultural Research Center, Sakha, Egypt. Axenic *S. platensis* culture was obtained from Algae Biotechnology Lab, Faculty of Science, Botany Department, Mansoura University.

### Preparation of *S. platensis* aqueous extract (SPAE)

Axenic *S. platensis* culture was kindly provided by Mansoura University Agal Culture Collection, then cultivated in sterilized Zarrouk’s liquid medium [34] as described by S Saad, MH Hussien, GS Abou-ElWafa, HS Aldesuquy and E Eltanahy [35] by using 10% culture then added to 5 L Zarrouk’s medium in a 10 L flask incubated at 26 ± 2 under 16:8 (light: dark) cool-white fluorescent at a light intensity equal to 3600 lx for 14 days. Then, the five liters were used as inoculum for 50 L vertical plastic bioreactors at the same conditions. After that, *S.* culture was filtered using a nylon mesh filter (100 μm diameter). Then, the harvested biomass was freeze-dried using lyophilizer for 48 h at -50 °C, obtaining the dry powder. *S. platensis* aqueous extract was prepared by homogenizing 5 g of the dry powder with 50 ml of distilled water and was ground until homogenized, then centrifuged for 10 min at 4000 rpm. The clear supernatant was collected and used as *S. platensis* aqueous extract (SPAE).

### Experimental design and treatments

Surface sterilization of homogeneous grains from the tested wheat cultivars was achieved by soaking them for 3 min in a 2.5% NaOCl solution, followed by multiple washes with sterilized distilled water. The sterilized grains of each cultivar were divided into two sets; the first set was presoaked for 18 h in sterilized distilled water, while the second set was presoaked in SPAE for the same duration. When they began soaking, the grains were drained for one hour and repeated every six hours for three draining sessions. The decision to utilize a 10% SPAE concentration and an 18-hour priming duration stemmed from initial germination trials, indicating that these parameters were optimal for promoting germination and bolstering drought resistance in wheat seedlings.

The experiment proceeded in The Greenhouse Facility, Faculty of Science, Mansoura University, Egypt. The presoaked grains were drilled at the beginning of the cultivation season (November 2021) within perforated plastic pots 30 cm in diameter and 45 cm in height. Each pot was packed with 10 kg clay-sandy soil (2: 1 w/w) to initially seed 15 grains in each pot till they were thinned, leaving only five uniform seedlings 30 days post-cultivation. Plants were allowed to grow under natural conditions: air temperature (15: 27 °C), relative humidity (40: 77%), and light intensity (10: 76 KLux) at midday time while irrigation was using tap water throughout the whole experimental period.

On day 45, the plants from each set (1st set, presoaked in distilled water (Dist. H_2_O), and 2nd set, presoaked in SPAE) were grouped into two subsets. The first subset was irrigated with tap water as a control treatment, whereas the second subset’s irrigation was stopped as a drought treatment. Then, the four treatments of each cultivar were control presoaked in distilled water (Cont), drought stress presoaked in distilled water (DS), control presoaked in *S. platensis* aqueous extract (SPAE-Cont), and drought stress presoaked in *S. platensis* aqueous extract (SPAE + DS). After 21 days, all treatments were 66 days old (heading stage), so flag leaf sampling was carried out to assess agronomy, pigment fractions, gas exchange, and carbohydrate content parameters. At the harvest stage (96 days old), the concerned wheat cultivars were evaluated for their yield and biochemical aspects (total carbohydrates and proteins) of the yielded grains.

### Assessment of leaf agronomy

To evaluate the morphological characteristics of the plants under both normal and stressful environments, we assessed the growth vigour of the flag leaf. Direct measurements captured certain agronomic attributes, such as fresh and dry biomass. Additionally, several traits were determined using established formulas:

Leaf area = length × breadth × 0.75 [36].

Leaf-specific area = leaf area/leaf dry mass [37].

Leaf water content = (Fresh mass - dry mass) / fresh mass [38].

Leaf succulence degree = (Leaf fresh mass - dry mass) / leaf area [39].

Leaf sclerophylly degree = Leaf dry mass/leaf area [39].

Leaf succulence quotient = leaf succulence degree/ leaf sclerophylly degree [40].

### Assessment of pigment fractions

The chlorophyll content of the plants under study was determined using the method described by [41]. For carotenoids, the protocols outlined by [42] and [43] were employed. A specified fresh mass of wheat leaves was finely ground in chilled 80% acetone, with a slight addition of MgCO_3_ to prevent pigment acidification. The resulting mixture was filtered, and the filtrate’s volume was made up using the same solvent. Subsequently, the absorbance (A) of the solution was recorded at three distinct wavelengths to deduce the concentration of the photosynthetic pigments, expressed in µg ml^− 1^ as follows:

Chlorophyll-a = 10.3 A_663_ − 0.918 A_644_.

Chlorophyll-b = 19.7 A_644_ − 3.87 A_663_.

Carotenoids = 5.02 A_480_.

The concentrations of the pigment fractions were ultimately denoted in µg mg^− 1^ of fresh mass. Several derived metrics, such as chlorophyll (a + b) as total chlorophylls, the ratio of chlorophyll (a/b), and the ratio of carotenoids to chlorophyll (a + b), were also determined. Notably, the chlorophyll stability index (CSI) was ascertained using the methodology proposed by [44] and is described as follows:

$$\begin{aligned} &CSI = \left[\right(Chlorophyll-a\hspace{0.17em}+\hspace{0.17em}b) \\& Drought / (Chlorophyll-a\hspace{0.17em}+\hspace{0.17em}b\left) Control\right] \times 100 \end{aligned}$$

### Assessment of leaf gas exchange

Using a portable gas exchange system (LCi, ADC BioScientific Ltd., Hoddesdon, UK), we assessed specific gas exchange parameters in situ around midday, utilizing the open flow mode. Leaves fully exposed to sunlight were aligned perpendicularly to the incoming rays, ensuring an average photosynthetically active radiation (PAR) of 700 µmol m^− 2^sec^− 1^, with a set chamber temperature of 28 °C and an ambient CO_2_ concentration (Ca) of 360 µmol mol^− 1^. Direct measurements captured parameters such as the photosynthesis rate (A), transpiration rate (E), stomatal conductance (gs), and the intercellular CO_2_ concentration (Ci). Furthermore, some gas exchange metrics were derived based on these direct measurements, as outlined in the subsequent equations:

Photosynthetic water use efficiency (pWUE) = A/ E [45].

Stomatal limitation (Ls) = 1- (Ci/ Ca) [46].

Mesophyll conductance (gm) = A/ Ci [47].

Mesophyll efficiency (ME) = Ci/ gs [48].

### Assessment of carbohydrates content

Flag leaves were extracted in 80% ethanol, and the resulting alcoholic extracts were analyzed for total soluble sugars (TSS) using the anthrone method [49]. Additionally, leaf samples were extracted in acid, trichloroacetic acid, and perchloric acid for the quantification of trehalose [50] and polysaccharides [51], respectively, via colourimetric anthrone assay.

### Assessment of yield attributes

To study yield attributes of the studied plants, some yield criteria were directly scored to study the plants’ yield attributes. These included plant height, shoot length, spike length, peduncle length, number of tillers/ plant, grains/ main spike, grains/ plant, spikes/ plant, spikelets/ main spike and spikelets/ plant, main spike mass, grain yield/ main spike, 100 kernel mass, biological yield (mass of the whole plant), straw yield (mass of the whole plant without grains), economic yield (mass of grains) and crop yield (mass of spikes with grains). Furthermore, other criteria could be calculated according to the following relations:

Evapotranspiration efficiency = water use efficiency for grain/ harvest index [52].

Water use efficiency for grain = grain yield/ total water added [53].

Water use efficiency for biomass = biomass yield/ total water added [53].

### Assessment of biochemical aspects of yielded grains

Total carbohydrates and proteins, the primary nutritional components in wheat grains, were quantified in yielded grain samples. For total carbohydrate determination, powdered grains were hydrolyzed in 2.5 N HCl by boiling for 3 h [49]. The mixture was neutralized with Na_2_CO_3_, then centrifuged, and the supernatant was analyzed colourimetrically with anthrone reagent at 630 nm. Protein extraction [54] involved homogenizing powdered grains in 0.05 mM Tris-HCl buffer (pH 9) and centrifuging the mixture. Bradford assay [55] was performed by mixing the protein-containing supernatant with Coomassie blue dye reagent and measuring absorbance at 595 nm after 1 h.

### Statistical analysis

In the current study, five replicates were taken from each treatment at the first sampling date (heading stage) and the second one (harvest stage) to evaluate leaf agronomy and yield attributes, respectively. Meanwhile, in the heading stage, only triplicate samples were used to assess the rest of the analyses, physio-biochemical assays of flag leaf (pigment fractions, gas exchange, and carbohydrate content), and biochemical assays of yielded grain (total carbohydrate and total protein content). A two-way completely randomized (2WCR) analysis of variance (ANOVA) using CoHort/ CoStat software version 6.311 (798 Lighthouse Ave. PMB 320, Monterey, CA, 93,940, USA) was employed to assess the single effect of each factor; factor (A) grain priming (Dist. H_2_O and SPAE), factor (B) watering level (normal irrigated ‘NI’, and drought ‘D’) and their interaction factor ‘A×B’ (Cont, DS, SPAE-Cont. and SPAE + DS) on the assessed parameters. This design was carried out at a significant level of *p* ≤ 0.05 with the LSD test. The data of the single effect of each factor were represented as the mean values in supplementary tables. Different superscript alphabetical letters refer to significant variation with the least significant difference (LSD) at *p* ≤ 0.05. The data of the interaction effect of grain priming and watering level were represented as mean values ± standard deviation in histogram figures with alphabetical letters on the error bar indicating significant differences with LSD at *p* ≤ 0.05.

## Result

All data of this study were analysed by two-way ANOVA then the Single effect of each grain priming and watering level on the assessed parameters for Shandawel 1 (Supplementary Table 1) and Sakha 95 (Supplementary Table 2) and their interactions in Supplementary Tables 3 and Supplementary Table 4, respectively. At the same time, each parameter was studied and represented in detail as follows.

### Alterations in leaf agronomy

**Fig. 1:**
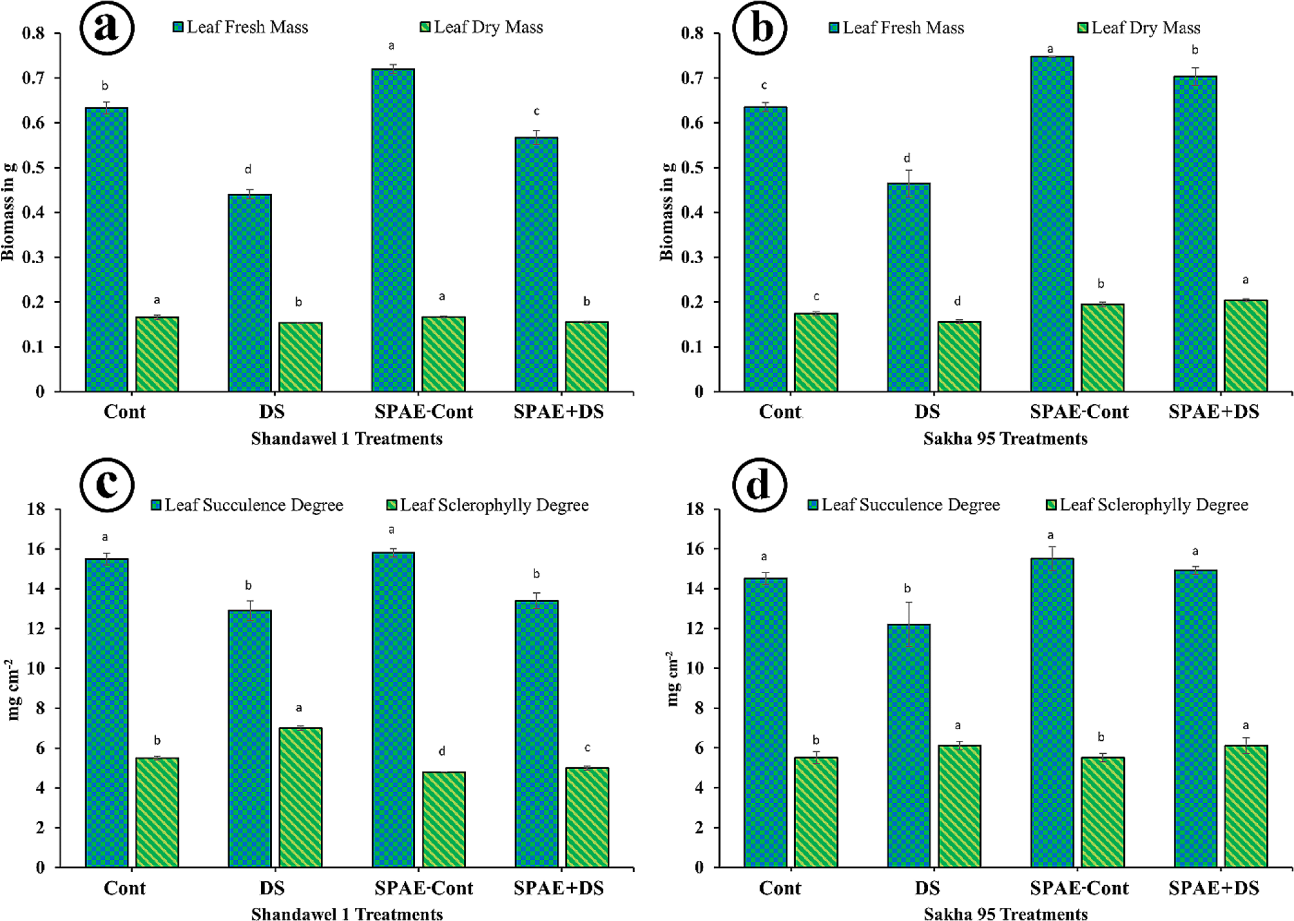
Effect of grain priming in *S. platensis* aqueous extract on flag leaf (**a**) biomass of Shandawel 1 cultivar, (**b**) biomass of Sakha 95 cultivar, (**c**) succulence degree, and sclerophylly degree of Shandawel 1 cultivar and (**d**) succulence degree, and sclerophylly degree of Sakha 95 cultivar

For Shandawel 1, the SPAE treatment notably enhanced the leaf fresh mass (Fig. 1-a), with a significant increase observed in the SPAE-Cont condition to 0.720 ± 0.010 g, 13.7% higher than the control (0.633 ± 0.014 g). The leaf fresh mass under drought stress (DS) decreased significantly to 0.440 ± 0.010 g but improved to 0.567 ± 0.015 g with the SPAE + DS treatment, indicating a 29% increase compared to DS alone. Similarly, Sakha 95 showed a 18% increase in leaf fresh mass (Fig. 1-b) with SPAE-Cont treatment (0.748 ± 0.00 g) compared to control (0.635 ± 0.01 g) and a 51.2% increase in SPAE + DS treatment (0.703 ± 0.02 g) compared to DS alone (0.465 ± 0.03 g). Shandawel 1’s leaf dry mass (Fig. 1-a) saw a slight increase with SPAE-Cont treatment to 0.167 ± 0.002 g, marginally higher than the control, and a minimal increase with SPAE + DS treatment compared to DS alone. Sakha 95 exhibited a 12% increase in leaf dry mass (Fig. 1-b) with SPAE-Cont treatment (0.195 ± 0.005 g) compared to Cont treatment and a significant 30.8% increase with SPAE + DS treatment (0.204 ± 0.003 g) compared to DS treatment.

Shandawel 1 showed non-significant increased leaf succulence (Fig. 1-c) with SPAE treatments; SPAE-Cont and SPAE + DS compared to the Cont and DS treatments, respectively. Conversely, leaf sclerophylly degree (Fig. 1-c) decreased significantly by 28.6% with SPAE + DS treatment (5.0 ± 0.1) compared to DS treatment and 4.2% compared to Cont treatment. Interestingly, the Cont treatment showed the least leaf sclerophylly degree. For Sakha 95, regarding leaf succulence degree (Fig. 1-d), the SPAE + DS treatment (14.9 ± 0.2 mg cm^− 2^) demonstrated a 22.1% significant increase compared to DS alone and a non-significant increase compared to the Cont treatment. The leaf sclerophylly degree (Fig. 1-d) is unchanged with SPAE treatments.

**Fig. 2:**
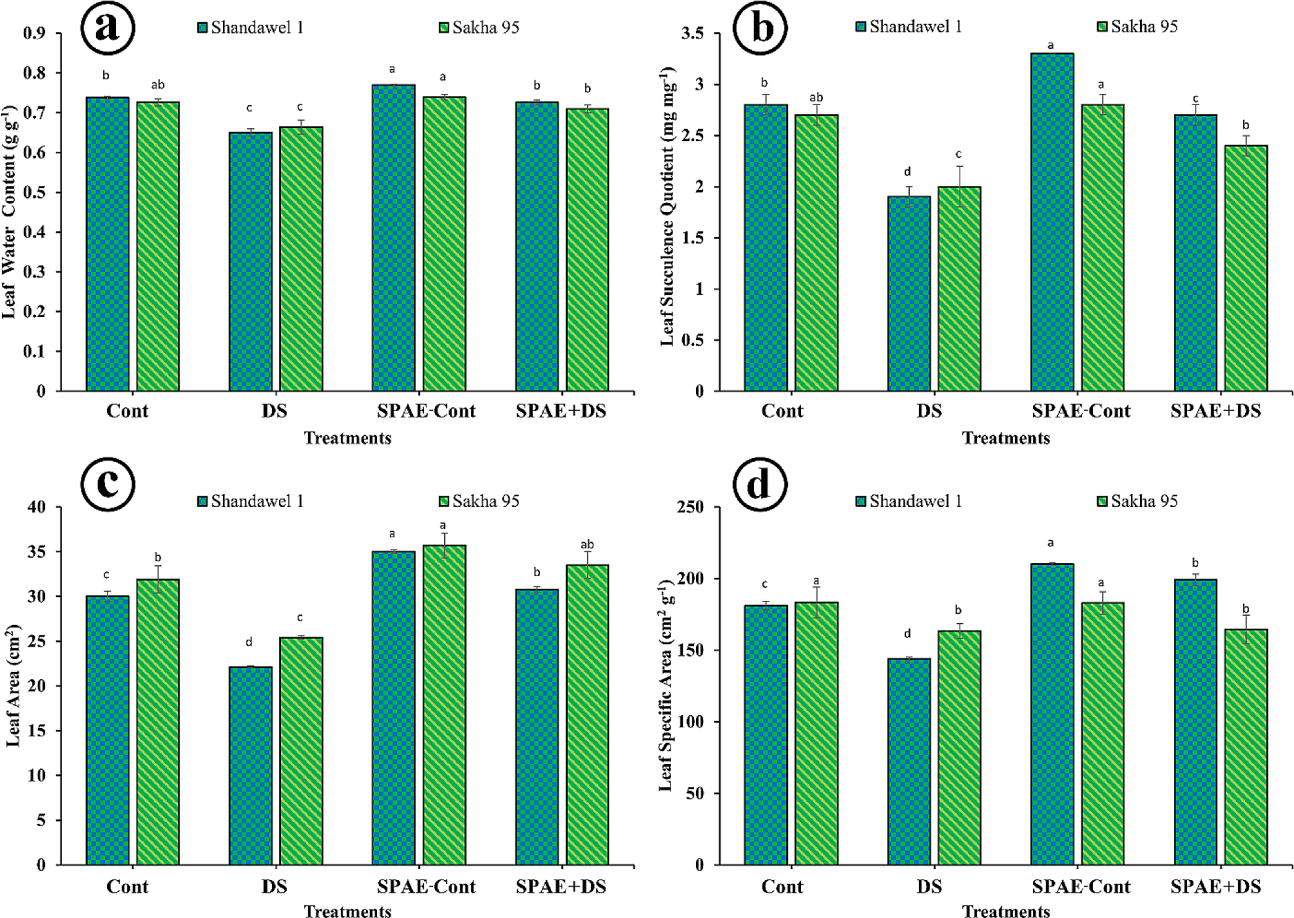
Effect of grain priming in *S. platensis* aqueous extract on flag leaf (**a**) water content of Shandawel 1 and Sakha 95 cultivars, (**b**) succulence quotient of Shandawel 1 and Sakha 95 cultivars, (**c**) area of Shandawel 1 and Sakha 95 cultivars and (**d**) specific area of Shandawel 1 and Sakha 95 cultivars

For Shandawel 1, the SPAE treatment significantly enhanced leaf water content as shown in Fig. 2-a to 0.769 ± 0.002 g g^− 1^, marking a 4.2% increase over the Cont treatment (0.738 ± 0.004 g g^− 1^). Under DS, leaf water content dropped to 0.650 ± 0.009 g g^− 1^, but with SPAE + DS treatment, it improved to 0.726 ± 0.007 g g^− 1^, indicating 11.7% enhancement compared to DS alone. The leaf succulence quotient (Fig. 2-b) in Shandawel 1 similarly benefited from SPAE treatment, with SPAE-Cont showing a 17.9% increase over control to 3.3 ± 0.0 mg mg^− 1^. The SPAE + DS treatment boosted the succulence quotient to 2.7 ± 0.1 mg mg^− 1^, substantially higher by 42.1% than DS alone. Leaf area Fig. 2-c responses also demonstrated SPAE’s efficacy, with SPAE-Cont treatment increasing leaf area by 16.7% over control to 35.0 ± 0.2 cm^2^ in Shandawel 1. The SPAE + DS treatment exhibited a notable 39.4% increase in leaf area compared to the DS treatment alone. For leaf-specific area (Fig. 2-d), SPAE-Cont treatment in Shandawel 1 significantly raised the metric to 210 ± 1 cm^2^ g^− 1^, 16% higher than control, with SPAE + DS showing a substantial 38.2% improvement over DS alone. In Sakha 95, leaf water content and succulence quotient with SPAE + DS showed a significant increase compared to DS alone and a non-significant decrease compared to the Cont treatment. Leaf area in Sakha 95 showed a significant increase with SPAE treatments, SPAE-Cont, and SPAE + DS compared to the Cont and DS treatments, respectively. The leaf specific area in Sakha 95 is non-significantly changed with SPAE treatments.

According to 2WCR, for Shandawel 1 (Supplementary Table 3), the interaction between grain priming and watering level factors was highly significant (*** at *p* ≤ 0.05) for sclerophylly degree, water content, area, and specific area, moderately significant for succulence quotient (** at *p* ≤ 0.05), lowly significant for fresh mass (* at *p* ≤ 0.05), and non-significant for dry mass and succulence degree (ns at *p* ≤ 0.05). For Sakha 95 (Supplementary Table 4), the interaction was highly significant (*** at *p* ≤ 0.05) for fresh mass and dry mass, lowly significant for the area (* at *p* ≤ 0.05), and non-significant for the other leaf agronomic parameters (ns at *p* ≤ 0.05).

### Alterations in pigment fractions

**Fig. 3:**
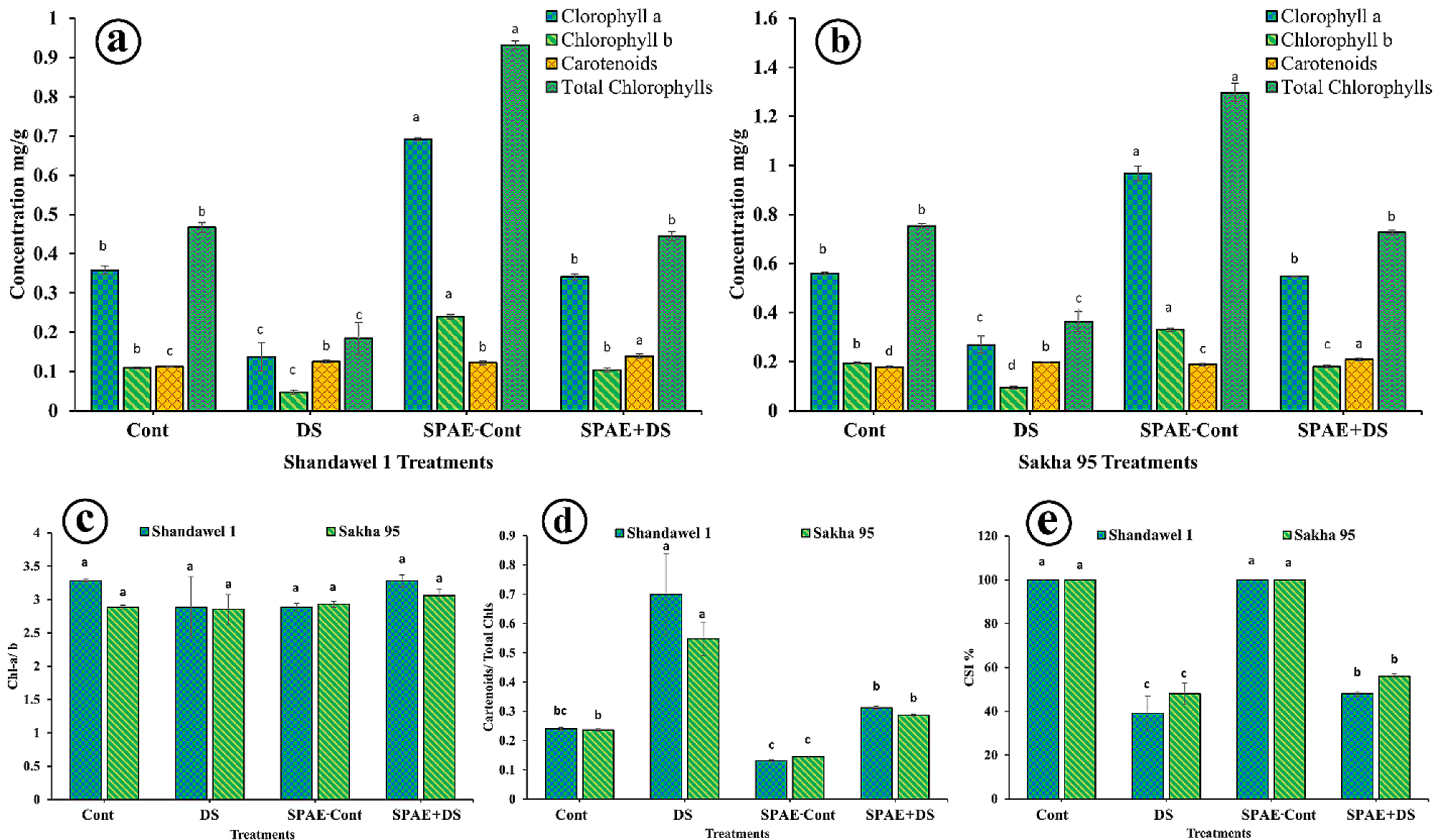
Effect of grain priming in *S. platensis* aqueous extract on flag leaf in heading stage (**a**) chlorophyll-a, chlorophyll-b, carotenoids, and total chlorophylls of Shandawel 1 cultivar, (**b**) chlorophyll-a, chlorophyll-b, carotenoids, and total chlorophylls content of Sakha 95 cultivar, (**c**) Chl-a/ b Shandawel 1 and Sakha 95 cultivars, (**d**) carotenoids/ total chlorophylls content of Shandawel and Sakha 95 cultivars, and (**e**) chlorophyll stability index (CSI%) of Shandawel 1 and Sakha 95 cultivars

For Shandawel 1, the SPAE-Cont treatment significantly increased the content of chlorophyll-a by 93%, chlorophyll-b by 120%, and total chlorophylls by 99% compared to the Cont treatment, as shown in Fig. 3-a and reflected in values of 0.692 ± 0.004, 0.240 ± 0.006, and 0.932 ± 0.010 mg g^− 1^, respectively. Interestingly, SPAE + DS treatment significantly increased the content of chlorophyll-a, chlorophyll-b, and total chlorophylls by 151%, 121%, and 142%, respectively, compared to DS treatment. In Sakha 95, the SPAE-Cont treatment led to the highest content of chlorophyll-a, chlorophyll-b, and total chlorophylls, with values of 0.967 ± 0.030, 0.330 ± 0.007, and 1.297 ± 0.037 mg g^− 1^, respectively (Fig. 3-b). In Shandawel 1, the SPAE + DS treatment showed the highest content of carotenoids of 0.139 ± 0.006 mg g^− 1^ compared to the other treatments (Fig. 3-a). Regarding Sakha 95, compared to the other treatments^,^ the SPAE + DS treatment resulted in the highest content of carotenoids of 0.209 ± 0.005 mg g^− 1^ (Fig. 3-b). The *S. platensis* treatments (SPAE-Cont and SPAE + DS) demonstrated a remarkable improvement in these parameters, emphasizing the potential beneficial effect of *S. platensis* in mitigating the negative impact of drought on pigment fractions wheat cultivars.

The chlorophyll-a/b ratio remained non-significantly changed across treatments in both cultivars (Fig. 3-c). In Shandawel 1, the SPAE + DS treatment with a chlorophyll stability index (CSI%) of 48 ± 1% (Fig. 3-e), representing 23%, over the DS treatment. The SPAE + DS treatment in Sakha 95 with a CSI % 56 ± 1% (Fig. 3-e)., representing 16%, compared to the DS treatment. It is essential to highlight that DS treatment generally led to significantly reduced chlorophyll content and CSI% in both cultivars while, on the other hand, inducing the carotenogenesis pathway, causing an increase in the carotenoids/ total chlorophylls values representing the highest values compared to the other treatments in both cultivars (Fig. 3-d).

According to 2WCR, for Shandawel 1 (Supplementary Table 3), the interaction between grain priming and watering level factors was highly significant (*** at *p* ≤ 0.05) for Chl-a, Chl-b, total Chls, moderately significant for carotenoids/ total chls (** at *p* ≤ 0.05), lowly significant for Chl-a/ b (* at *p* ≤ 0.05), and non-significant for CSI% (ns at *p* ≤ 0.05). The interaction for Sakha 95 (Supplementary Table 4) was highly significant (*** at *p* ≤ 0.05) for Chl-b, total Chls, moderately significant for Chl-a, and carotenoids/ total chls (** at *p* ≤ 0.05), lowly significant for CSI% (* at *p* ≤ 0.05), and non-significant for carotenoids, and Chl-a/ b (ns at *p* ≤ 0.05).

### Alterations in gas exchange

**Fig. 4:**
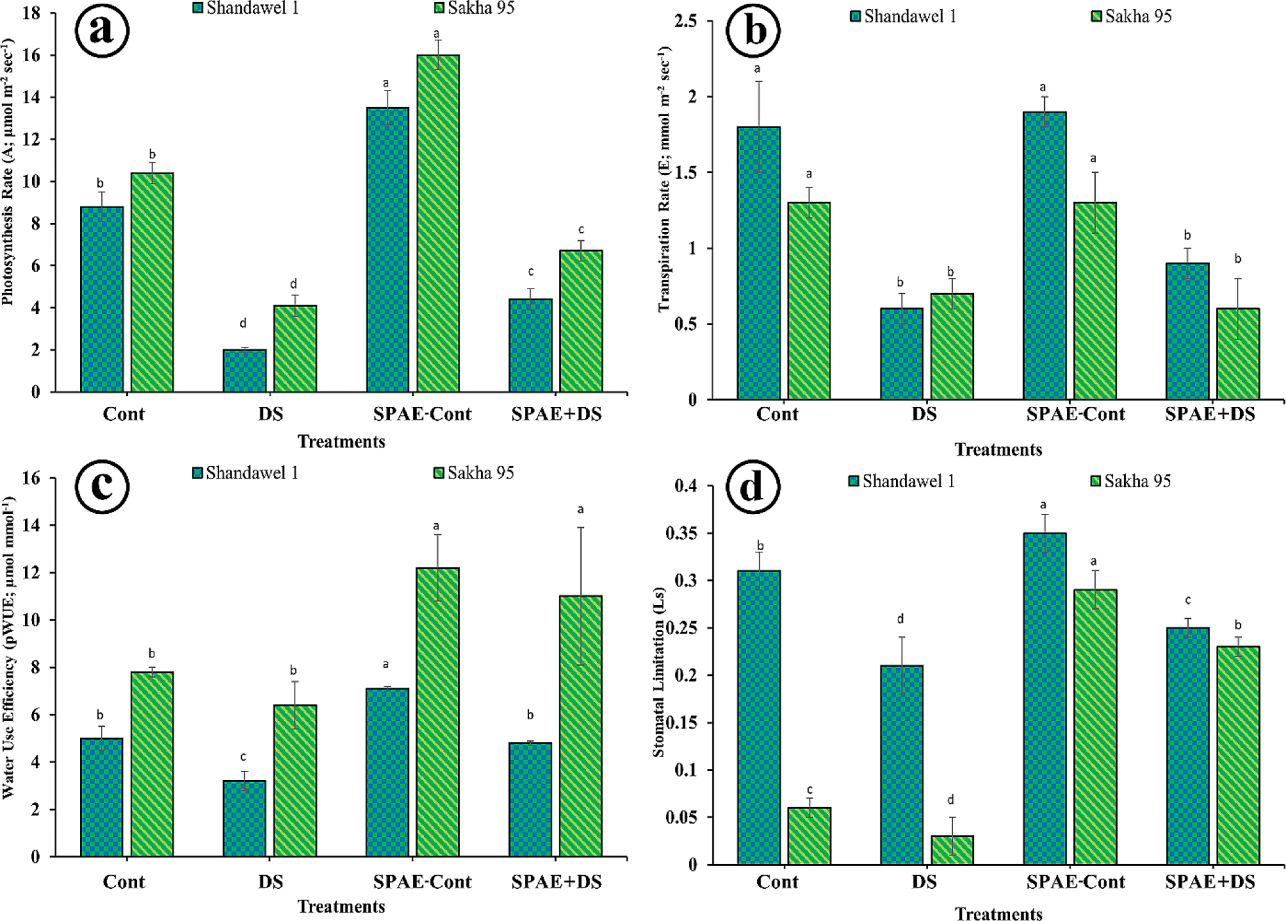
Effect of grain priming in *S. platensis* aqueous extract on Shandawel 1 and Sakha 95 cultivars: (**a**) photosynthesis rate, (**b**) transpiration rate, (**c**) water use efficiency, and (**d**) stomatal limitation

For Shandawel 1the SPAE-Cont treatment showed the highest photosynthetic rate (Fig. 4-a) at 13.5 ± 0.8 µmol m^− 2^ sec^− 1^, significantly surpassing the control (8.8 ± 0.7 µmol m^− 2^ sec^− 1^), SPAE + DS (4.4 ± 0.5 µmol m^− 2^ sec^− 1^), and DS (2 ± 0.1 µmol m^− 2^ sec^− 1^) treatments. Notably, the photosynthetic rate under DS treatment plummeted by 340% compared to the control, highlighting the severe impact of drought stress. However, the SPAE + DS treatment improved the photosynthetic rate by 50% over DS alone. In the case of Sakha 95, similar trends were observed, with SPAE-Cont treatment achieving the highest photosynthesis rate at 16 ± 0.7 µmol m^− 2^ sec^− 1^. The ranking of treatments by photosynthetic rate followed with Cont (10.4 ± 0.5 µmol m^− 2^ sec^− 1^), SPAE + DS (6.7 ± 0.5 µmol m^− 2^ sec^− 1^), and DS (4.1 ± 0.5 µmol m^− 2^ sec^− 1^). The control condition’s photosynthetic rate decreased by 136% under DS, illustrating the detrimental effect of drought stress. However, the SPAE-Cont treatment significantly boosted the photosynthesis rate by 54% compared to the control. Moreover, the SPAE + DS treatment exhibited a 50% higher rate than the DS treatment alone. Regarding the transpiration rate, as shown in Fig. 4-b, the rate significantly decreased by the DS effect in the two wheat cultivars while the SPAE-Cont showed a non-significant effect compared to the control and even for SPAE + DS so, there is no major differences were observed in the case of using *S. platensis* aqueous extract, but DS resulted in lower rates in both cultivars.

In Shandawel 1, SPAE-Cont treatment significantly outperformed other treatments in pWUE, as shown in Fig. 4-c, recording the highest efficiency at 7.1 ± 0.1 µmol mmol^− 1^, a 42% increase over the control, and SPAE + DS treatment showing a 50% improvement over DS treatment. Similarly, SPAE treatments markedly improved Ls (Fig. 4-d), with SPAE-Cont achieving a 13% higher value than the control and SPAE + DS demonstrating a 19% increase over DS alone. For Sakha 95, SPAE-Cont and SPAE + DS also led in pWUE, with significant gains over control and DS treatments, and similarly exhibited a substantial uplift in Ls, nearly quadrupling the control’s value and significantly outpacing DS treatment.

**Fig. 5:**
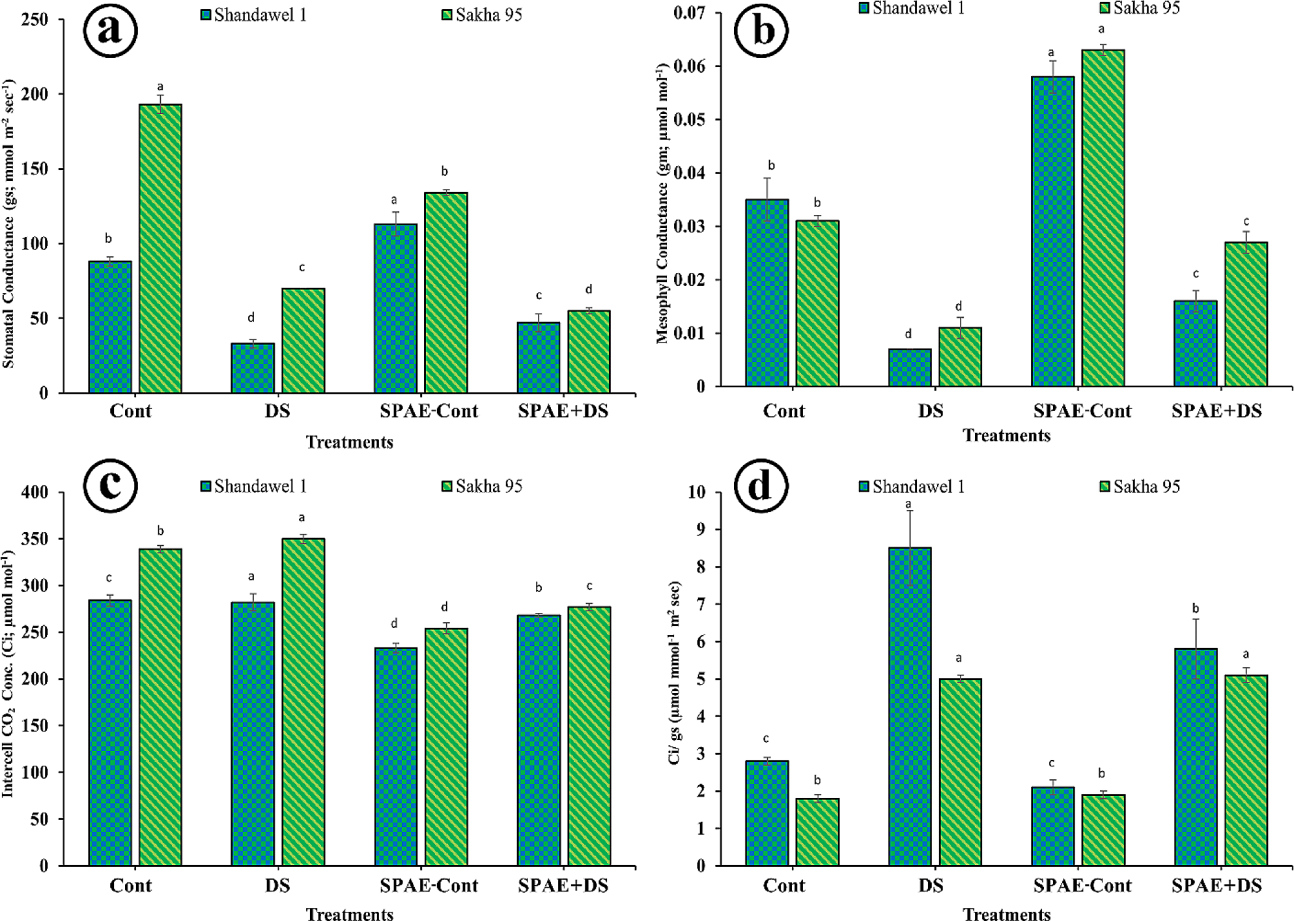
Effect of grain priming in *S. platensis* aqueous extract on Shandawel 1 and Sakha 95 cultivars: (**a**) stomatal conductance, (**b**) mesophyll conductance, (**c**) intercellular CO_2_ concentration, and (**d**) intercell CO_2_ to stomatal conductance (***Ci/ gs)***

According to 2WCR in Shandawel 1, SPAE treatment notably increased gs (Fig. 5-a), with SPAE-Cont showing a 28.4% enhancement over control, and SPAE + DS improving gs by 42.4% DS. Similarly, SPAE treatment significantly raised gm (Fig. 5-b) in Shandawel 1, with SPAE-Cont and SPAE + DS treatments leading to substantial increases over control and DS conditions, respectively. Ci as shown in Fig. 5-c and Ci/ gs and as shown in Fig. 5-d revealed that SPAE treatments could modulate Ci under drought stress, with SPAE-Cont and SPAE + DS treatments affecting Ci differently in comparison to DS. For Sakha 95, trends were analogous, with SPAE treatments improving gs and gm and effectively modulating Ci and Ci/gs ratios.

Utilizing 2WCR for Shandawel 1 as shown in Supplementary Table 3, the interaction between grain priming and watering level factors was moderately significant for A and gm (** at *p* ≤ 0.05), lowly significant for Ci/ gs (* at *p* ≤ 0.05), and non-significant for the other leaf gas exchange parameters (ns at *p* ≤ 0.05). The interaction for Skaha 95 (Supplementary Table 4) was highly significant (*** at *p* ≤ 0.05) for A, gs, and gm, as well as non-significant for the other leaf gas exchange parameters (ns at *p* ≤ 0.05).

### Alterations in carbohydrate content

**Fig. 6:**
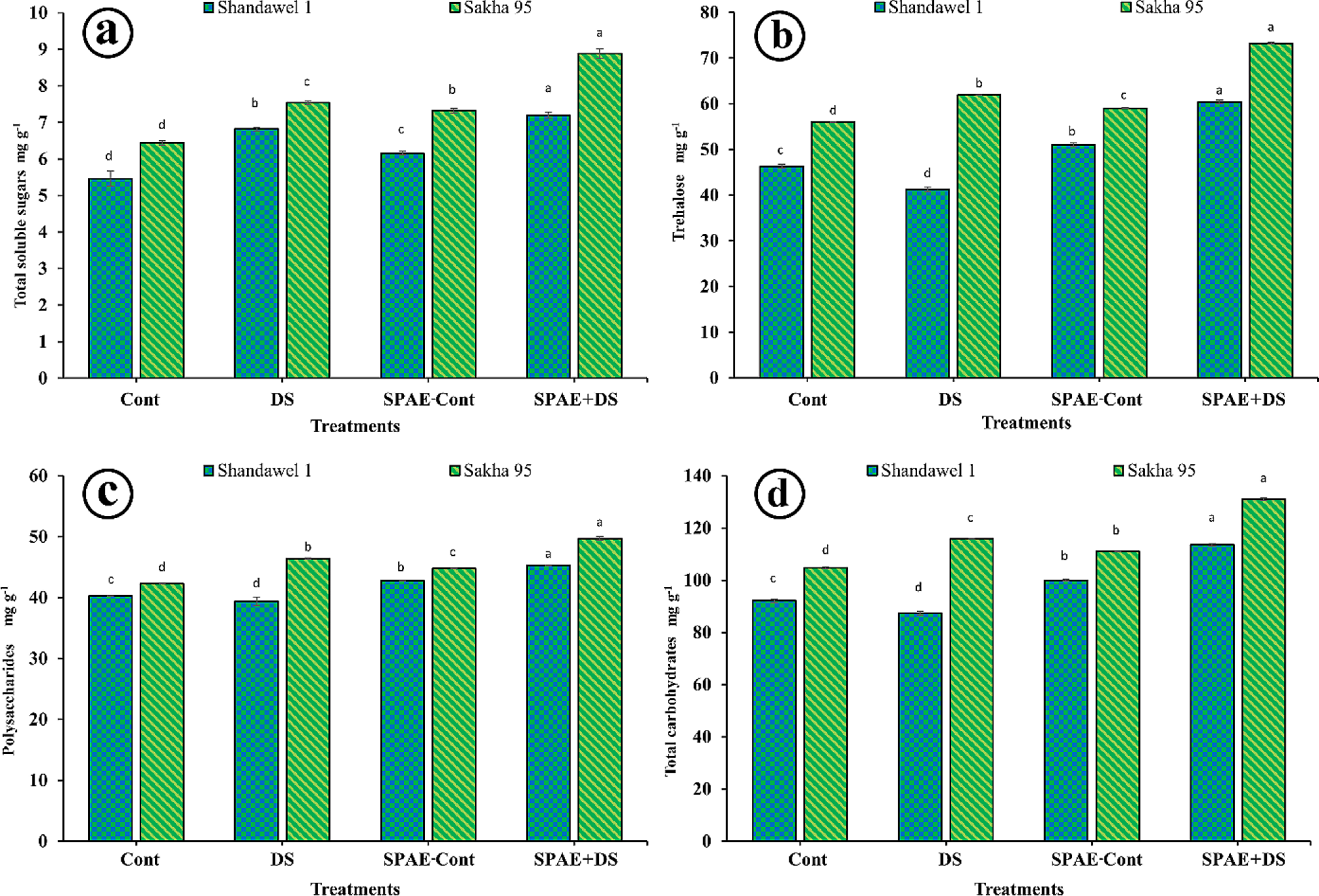
Effect of grain priming in *S. platensis* aqueous extract on flag leaf in heading: (**a**) total soluble sugars, (**b**) trehalose, (**c**) polysaccharides, and (**d**) total carbohydrates, content of Shandawel 1 and Sakha 95 cultivars

For Shandawel 1, SPAE + DS resulted in a significant increase in TSS content (7.91 ± 0.08 mg g^− 1^) as shown in Fig. 6-a, compared to all other treatments, including the control (5.46 ± 0.21 mg g^− 1^), DS alone (6.82 ± 0.05 mg g^− 1^), and SPAE-Cont (6.15 ± 0.05 mg g^− 1^). This indicates a 45%, 16%, and 29% increase compared to the Cont, DS, and SPAE-Cont, respectively. Similar trends were observed in Sakha 95, with the SPAE + DS treatment resulting in the highest TSS content (8.88 ± 0.13 mg g^− 1^), a 38%, 18%, and 21% increase compared to Cont treatment, DS, and SPAE-Cont treatments, respectively. Concerning trehalose content (Fig. 6-b), the SPAE + DS treatment again resulted in a significant increase in both Shandawel 1 (60.4 ± 0.4 mg g^− 1^) and Sakha 95 (73.2 ± 0.2 mg g^− 1^), compared to all other treatments. For Shandawel 1, this was an 18% significant increase compared to SPAE-Cont and a 47% significant increase compared to the DS treatment alone. In Sakha 95, the SPAE + DS treatment resulted in an 18% and 24% increase compared to the DS alone and SPAE-Cont treatments, respectively.

The polysaccharide content (Fig. 6-c) showed a similar trend, with the SPAE + DS treatment resulting in the highest content in both Shandawel 1 (45.3 ± 0.1 mg g^− 1^) and Sakha 95 (49.7 ± 0.3 mg g^− 1^). This represented a 6%, 15%, and 12.4% significant increase compared to SPAE-Cont, DS alone, and the control in Shandawel 1, and a 7%, 11%, and 17% significant increase in Sakha 95, respectively. Moreover, the total carbohydrates were significantly higher in the SPAE + DS treatment compared to all other treatments for both cultivars. For Shandawel 1, the SPAE + DS treatment (113.6 ± 0.4 mg g^− 1^) was 14%, 30%, and 23% higher than SPAE-Cont, DS alone and the control, respectively. Similarly, in Sakha 95, the SPAE + DS treatment (131.7 ± 0.6 mg g^− 1^) was 14%, 19%, and 26% higher compared to DS alone, SPAE-Cont, and the control, respectively as shown in Fig. 6-d.

According to 2WCR ANOVA (Supplementary Tables 3 and 4), the interaction between grain priming and watering level factors had the same high effect in both cultivars (*** at *p* ≤ 0.05) for trehalose and total carbohydrates. The interaction in Shandawel 1 was highly significant (*** at *p* ≤ 0.05) for polysaccharides and lowly significant for TSS (* at *p* ≤ 0.05). Moreover, the interaction in Skaha 95 was moderately significant for TSS and polysaccharides (** at *p* ≤ 0.05).

### Alterations in yield attributes

**Fig. 7:**
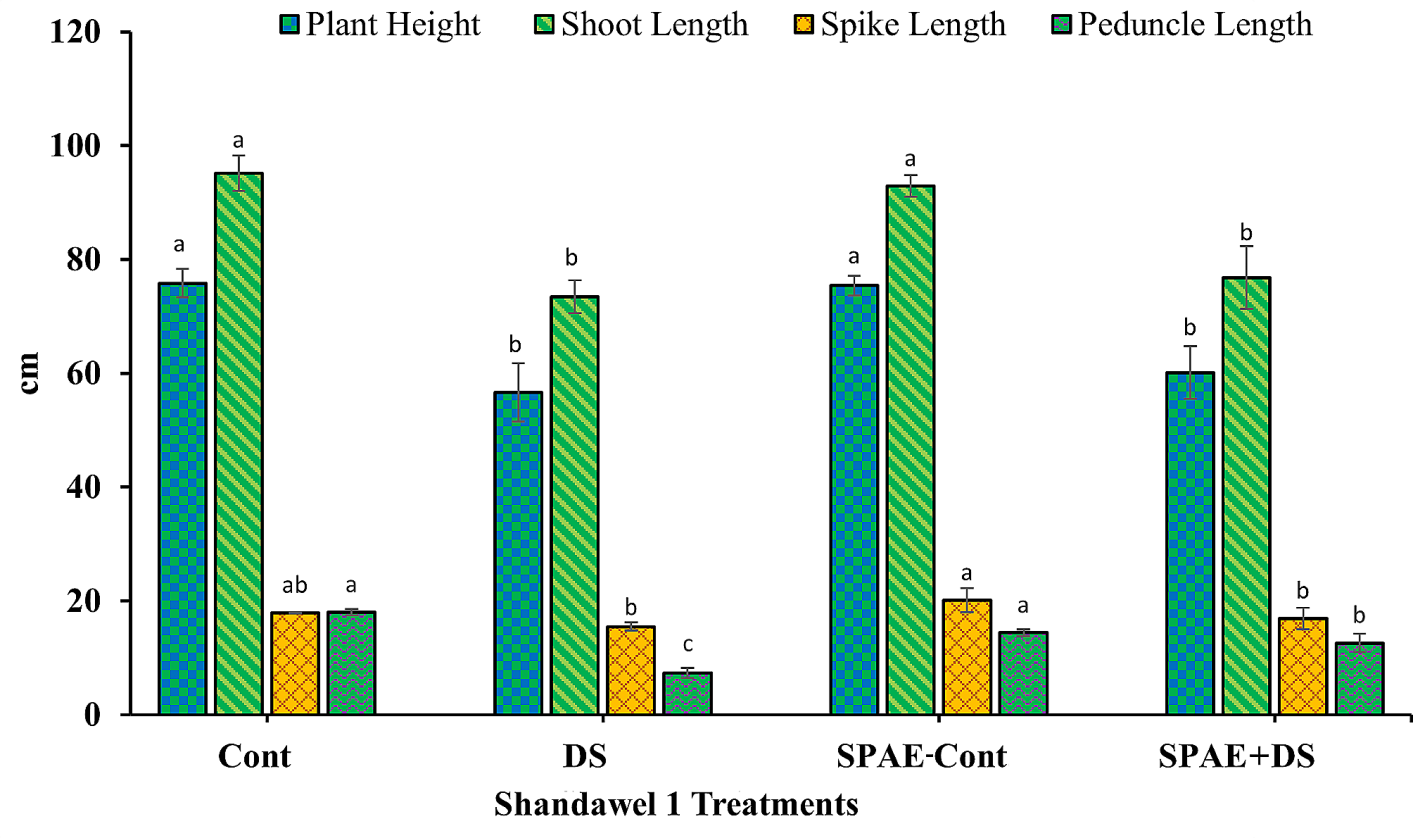
Effect of grain priming in *S. platensis* aqueous extract on plant height, shoot length, spike length, and peduncle length of Shandawel 1 cultivar

In Shandawel 1 (Fig. 7), the plant height was 75.8 ± 2.5 cm, significantly decreasing by 25% under DS to 56.6 ± 5.1 cm. The SPAE-Cont treatment, however, showed negligible height reduction, recording 75.4 ± 1.7 cm. The SPAE + DS treatment resulted in a 21% decrease in height compared to the SPAE-Cont treatment but still held a 6% advantage over DS alone. Concerning the shoot length of Shandawel 1, DS treatment caused a significant contraction of 22.8% from the control’s length (95.1 ± 3.1 cm). The SPAE-Cont treatment yielded a minor decrease of 2.3% (92.9 ± 1.9 cm). The SPAE + DS treatment resulted in a substantial 17.4% decrease from SPAE-Cont but offered a 4.6% advantage over DS alone. Spike length in Shandawel 1 reduced by 13% under DS from the control’s 17.9 ± 0.1 cm, which is still non-significant, while SPAE-Cont resulted in a notable 12.3% enhancement (20.1 ± 2.1 cm). The SPAE + DS treatment led to a 15.9% reduction from SPAE-Cont, but it outperformed DS alone by 9%. Regarding the peduncle length of Shandawel 1, DS caused a significant decrease of 59% from the control’s 18.0 ± 0.6 cm. The SPAE-Cont treatment showed a nonsignificant reduction of 20% but was 97%, significantly higher than DS. The SPAE + DS treatment resulted in a 30% decrease from the control, while it was still 73% higher than DS alone.

**Fig. 8:**
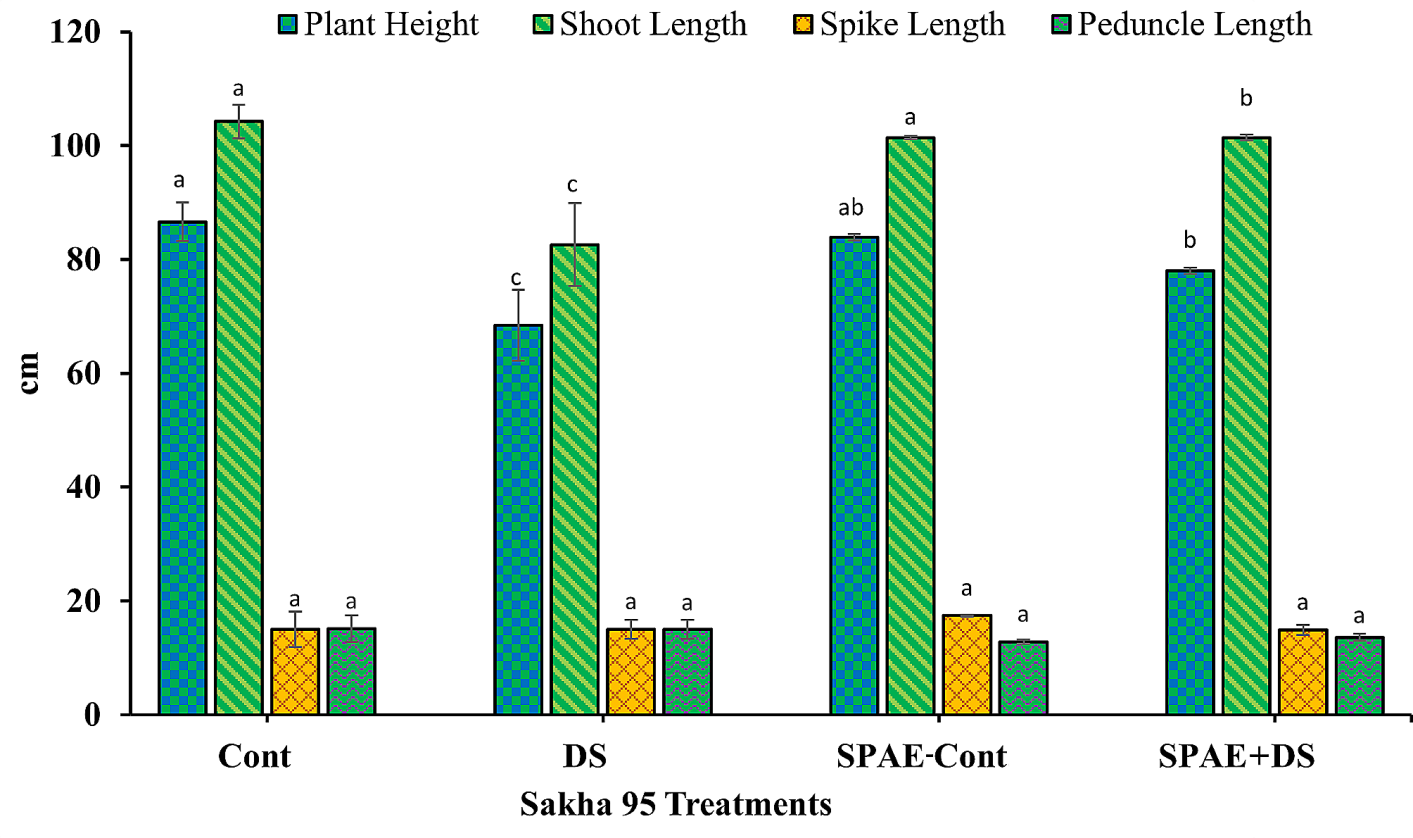
Effect of grain priming in *S. platensis* aqueous extract on plant height, shoot length, spike length and peduncle length of Sakha 95 cultivar

Sakha 95 exhibited similar patterns as shown in Fig. 8: a decrease of 21% in plant height from 86.6 ± 3.4 cm under DS and 3% under SPAE-Cont (83.9 ± 0.6 cm) from the Cont treatment. The SPAE + DS treatment saw a reduction of 7.0% from SPAE-Cont, yet it was still 14% higher than DS alone. Sakha 95 shoot length also showed a decrease of 21% under DS from the control’s 104.2 ± 2.9 cm and a 2.7% reduction under SPAE-Cont. The SPAE + DS treatment demonstrated a 9% decrease from SPAE-Cont, yet it was 12% higher than DS alone. In Sakha 95, spike length remained unchanged under DS (15.0 ± 1.7 cm) compared to the control, while SPAE-Cont showed a 16% non-significant increase (17.4 ± 0.2 cm). The SPAE + DS treatment showed a 14% non-significant decrease from SPAE-Cont and only a marginal 1% decrease from DS alone but non-significantly. In Sakha 95, peduncle length remained relatively constant under DS (15.0 ± 1.7 cm) compared to the control. It decreased non-significantly by 15% under SPAE-Cont, while the SPAE + DS treatment showed a 10% non-significant decrease from the control but a 6% non-significant increase from SPAE-Cont.

**Fig. 9:**
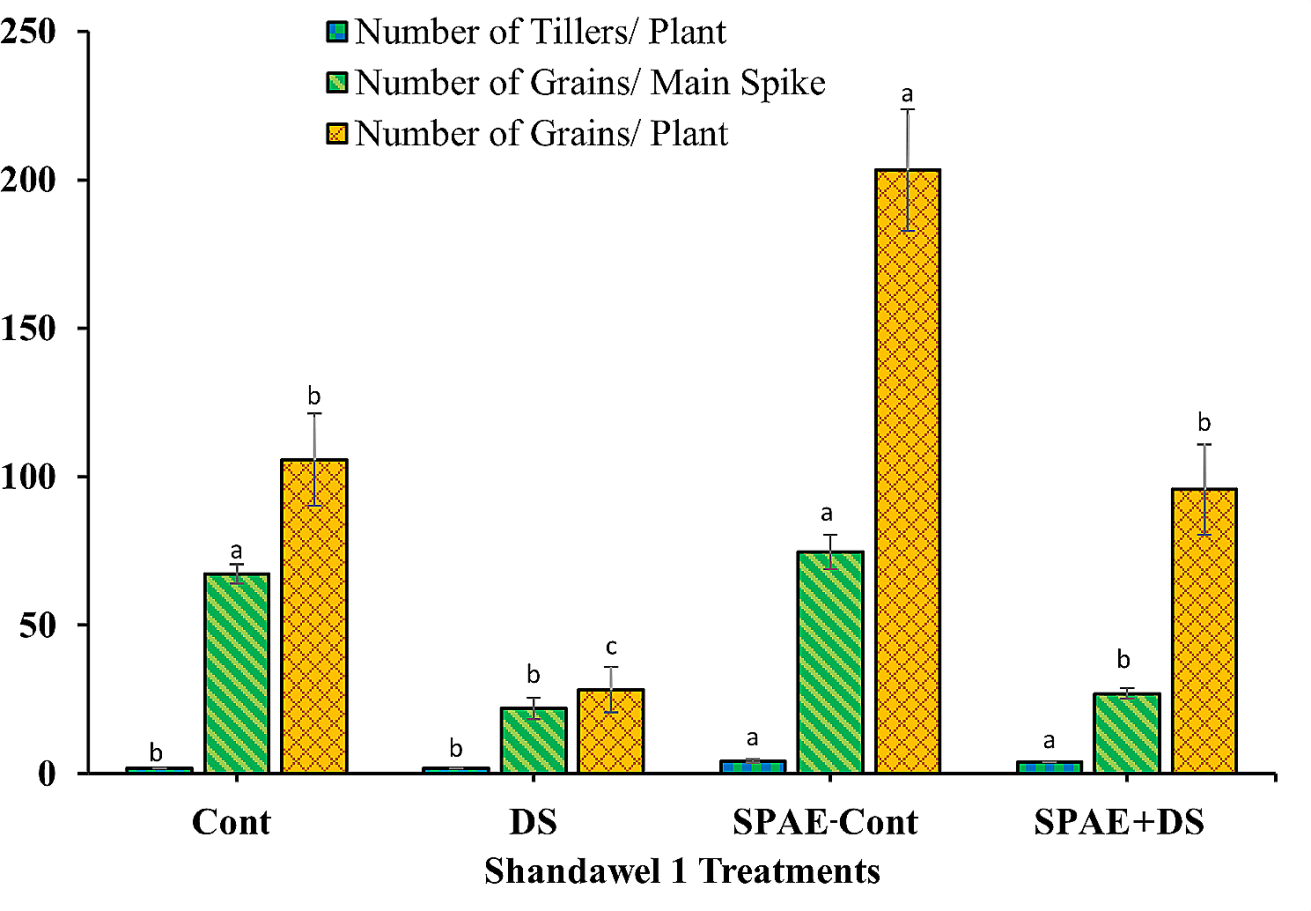
Effect of grain priming in *S. platensis* aqueous extract on number of tillers/ plant, number of grains/ main spike and number of grains/ plant of Shandawel 1 cultivar

The number of tillers per plant in Shandawel 1, as shown in Fig. 9, exhibits a mean of 2.0 tillers/ plant for Cont treatment, which remained constant under DS treatment. However, the SPAE-Cont treatment significantly increased the number of tillers/ plant by 115% (4.3 ± 0.6 tillers/ plant). While the SPAE + DS treatment resulted in a slight non-significant reduction in the number of tillers/ plant (4.0 tillers per plant) compared to SPAE-Cont, it still held a 100% advantage over the Cont and the DS treatments. The number of grains per main spike in Shandawel 1 significantly declined under DS (22.0 ± 3.6), recording a significant decrease of 67% from the Cont treatment (67.3 ± 3.2). The SPAE-Cont treatment increased considerably by 11% to 74.7 ± 5.9 compared to the Cont treatment, further demonstrating the beneficial role of *S. platensis*. The SPAE + DS treatment, however, saw a 64% decrease from SPAE-Cont, yet it was 23% higher than the DS treatment, reinforcing the role of *S. platensis* as a potential drought stress mitigator. Regarding the number of grains per plant in Shandawel 1, there was a drastic reduction of 73% under DS (28.3 ± 7.6) from the Cont treatment (105.7 ± 15.5). The SPAE-Cont treatment recorded an impressive increase (203.3 ± 20.5) of 92% compared to the Cont treatment, which is a promising result for final crop yield. The SPAE + DS treatment led to a 238% significant increase in the number of grains/ plant compared to DS, suggesting *S. platensis*’s potential as a stress alleviator.

**Fig. 10:**
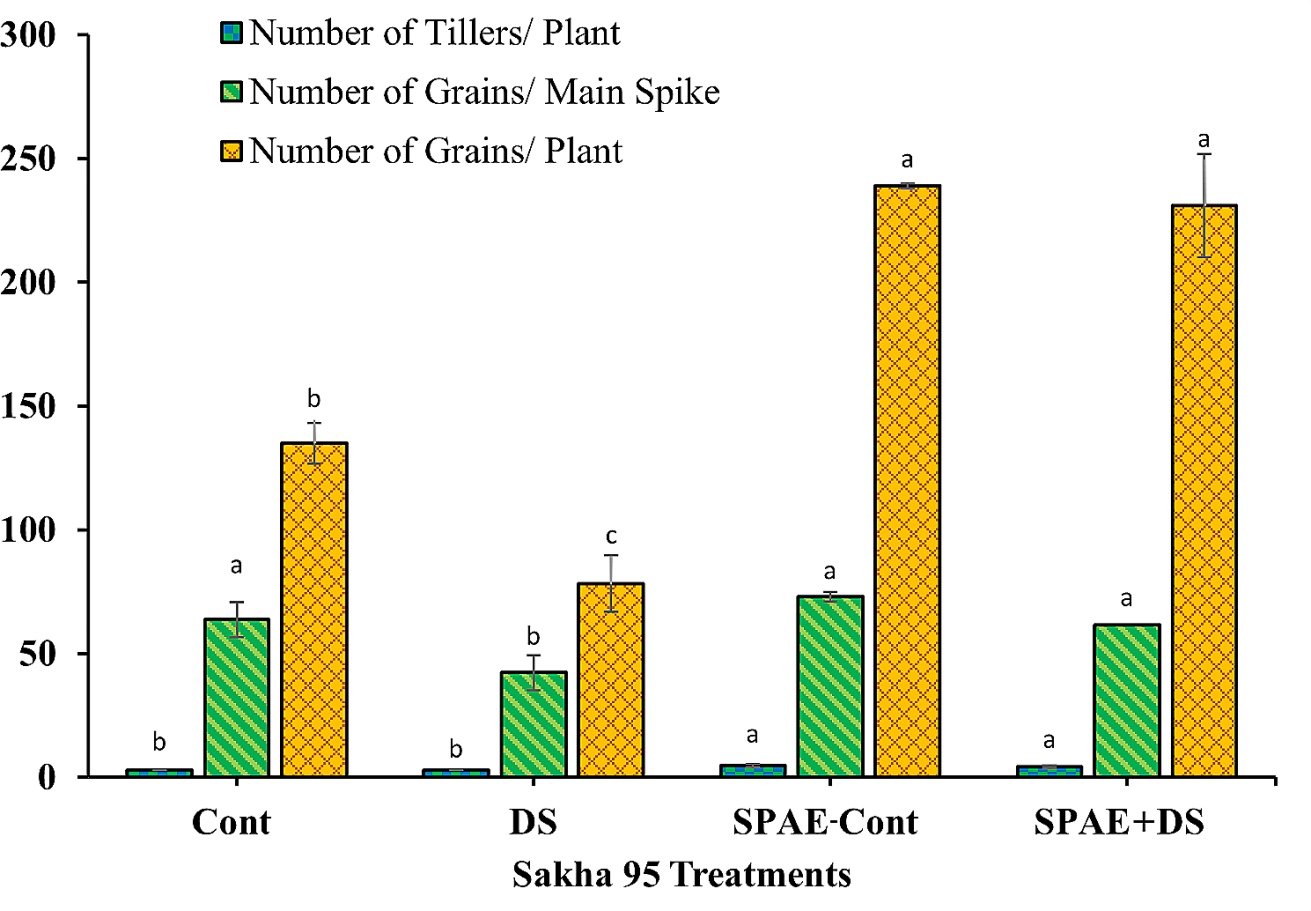
Effect of grain priming in *S. platensis* aqueous extract on number of tillers/ plant, number of grains/ main spike and number of grains/ plant of Sakha 95 cultivar

The tolerant cultivar, Sakha 95, displayed similar patterns (Fig. 10): the number of tillers per plant remained unchanged under DS and saw a 57% significant increase (4.7 ± 0.6) under the SPAE-Cont treatment compared to the Cont treatment in the Cont treatment (3.0 ± 0.0). The SPAE + DS treatment had 43% more tillers per plant (4.3 ± 0.6) than DS treatment. The number of grains per main spike significantly decreased by 34% under DS from the Cont treatment (63.7 ± 7.1) and significantly increased by 15% under SPAE-Cont. The number of grains per main spike in the SPAE + DS treatment (61.7 ± 7.5) recorded only a marginal 15% decrease compared to SPAE-Cont and a 46% significant increase compared to DS. The number of grains per plant in Sakha 95 was reduced by 42% under DS, while the SPAE-Cont treatment showed a 77% increase compared to the Cont tretment. Despite a 3% non-significant reduction under the combined SPAE + DS treatment compared to the SPAE-Cont, it still had almost twofold more grains per plant than DS alone, underlining the protective role of *S. platensis*.

**Fig. 11:**
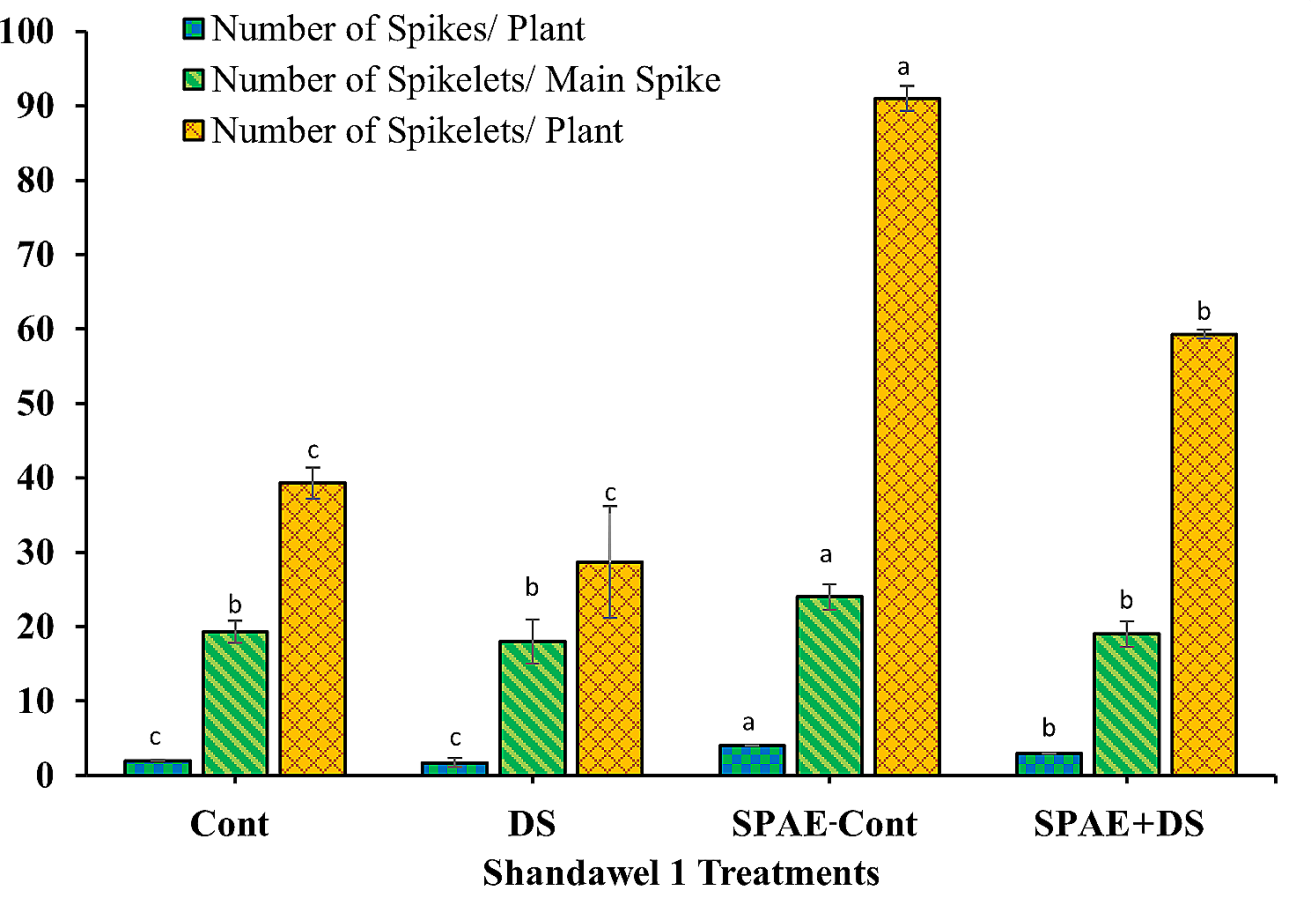
Effect of grain priming in *S. platensis* aqueous extract on number of spikes/ plant, number of spikelets/ main spike and number of spikelets/ plant of Shandawel 1 cultivar

In the sensitive cultivar Shandawel 1 (Fig. 11), the control treatment (Cont) exhibited an average of 2.0 ± 0.0 spikes per plant, non-significantly dropping by 15% under the DS condition, recording 1.7 ± 0.6 spikes per plant. This decrease, however, was mitigated with the SPAE-Cont treatment, demonstrating an enhanced number of spikes per plant (4.0 ± 0.0). The combined application of SPAE and DS resulted in a 25% decline in the number of spikes per plant (3.0 ± 0.0) compared to the SPAE-Cont treatment but still presented a considerable 50% and 76% increment over the Cont and DS treatments, respectively. These findings point towards the stress-alleviating capabilities of *S. platensis*, especially under drought-stress conditions. In terms of the number of spikelets per main spike, a reduction of 7% was recorded under DS from the control’s 19.3 ± 1.5. Meanwhile, SPAE-Cont demonstrated a remarkable 24% enhancement (24.0 ± 1.7), further advocating the beneficial properties of *S. platensis*. The combined SPAE + DS treatment led to a 20.8% decline from SPAE-Cont, yet it still showed a slight 6% advantage over the DS treatment alone. As for the number of spikelets per plant, the DS treatment caused a substantial 27% non-significant decrease from the control’s 39.3 ± 2.1. The SPAE-Cont treatment, on the other hand, showcased a significant increase, recording 91.0 ± 1.7 spikelets per plant - an impressive 132% enhancement from the control. The combined SPAE + DS treatment resulted in a significant 35% reduction from the SPAE-Cont but offered a 51% and 106% improvement over the Cont and DS treatments, respectively.

**Fig. 12:**
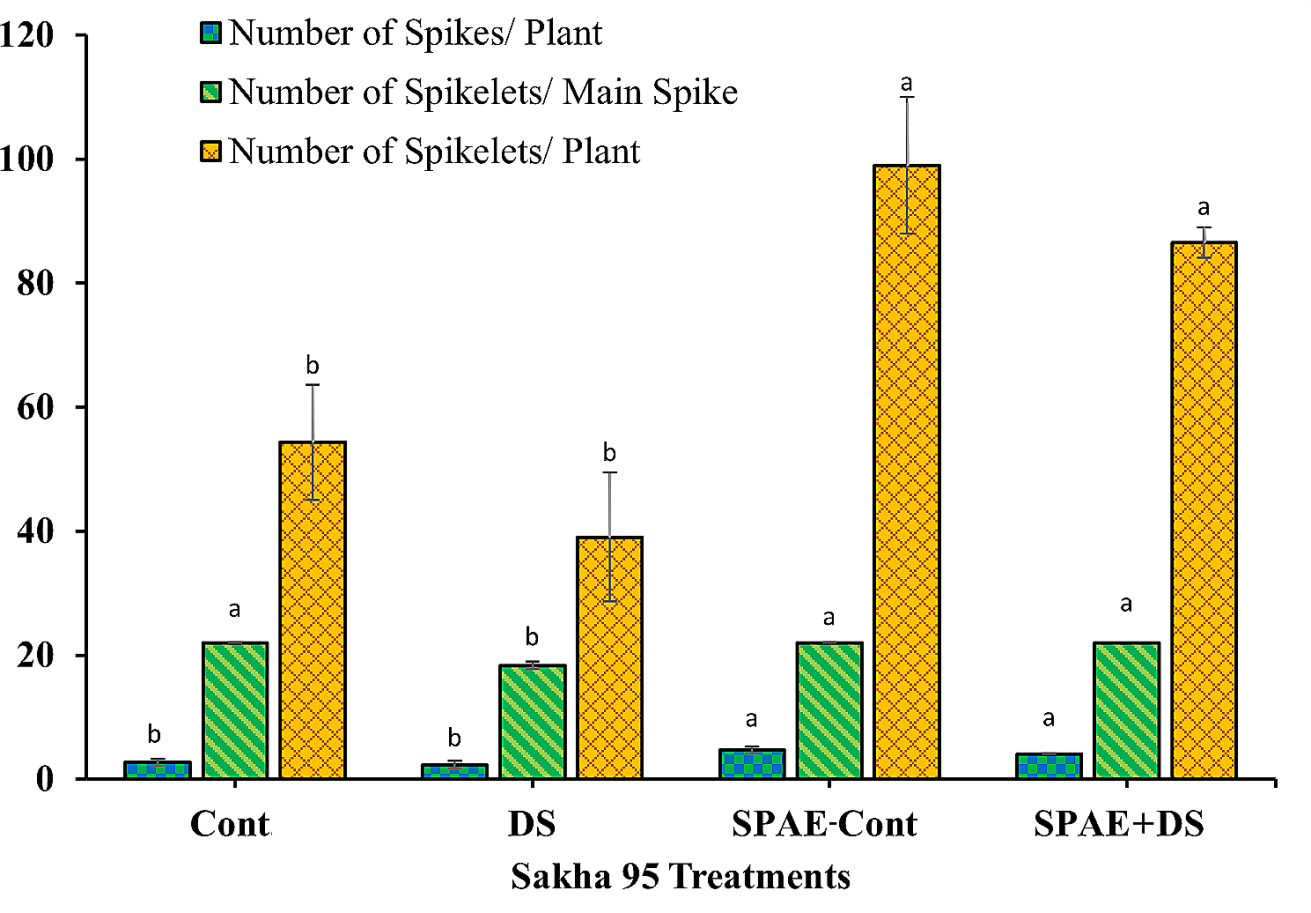
Effect of grain priming in *S. platensis* aqueous extract on number of spikes/ plant, number of spikelets/ main spike and number of spikelets/ plant of Sakha 95 cultivar

The tolerant cultivar, Sakha 95, demonstrated similar trends (Fig. 12). A non-significant decrease of 15% from 2.7 ± 0.6 spikes per plant under DS and a 74% significant increase under SPAE-Cont (4.7 ± 0.6 spikes per plant) from the control were recorded. The combined SPAE + DS treatment exhibited a minor 15% non-significant decrease from SPAE-Cont, yet it was still 48% and 73% significantly higher than the Cont and DS treatments, respectively, reiterating the potential mitigating effects of *S. platensis* against drought stress. When observing the number of spikelets per main spike, the DS treatment led to a decline of 17% from the control’s 22.0 ± 0.0, while the number remained stable under SPAE-Cont and SPAE + DS treatments, hence pointing towards the protective role of *S. platensis*. Lastly, concerning the number of spikelets per plant, Sakha 95 showed a non-significant decrease of 28% under DS from the control’s 54.3 ± 9.3, and an 82% significant increase under SPAE-Cont The combined SPAE + DS treatment displayed a non-significant reduction of 13% from SPAE-Cont, yet it was 59% and 122% significantly higher than the Cont and DS treatments, respectively.

**Fig. 13:**
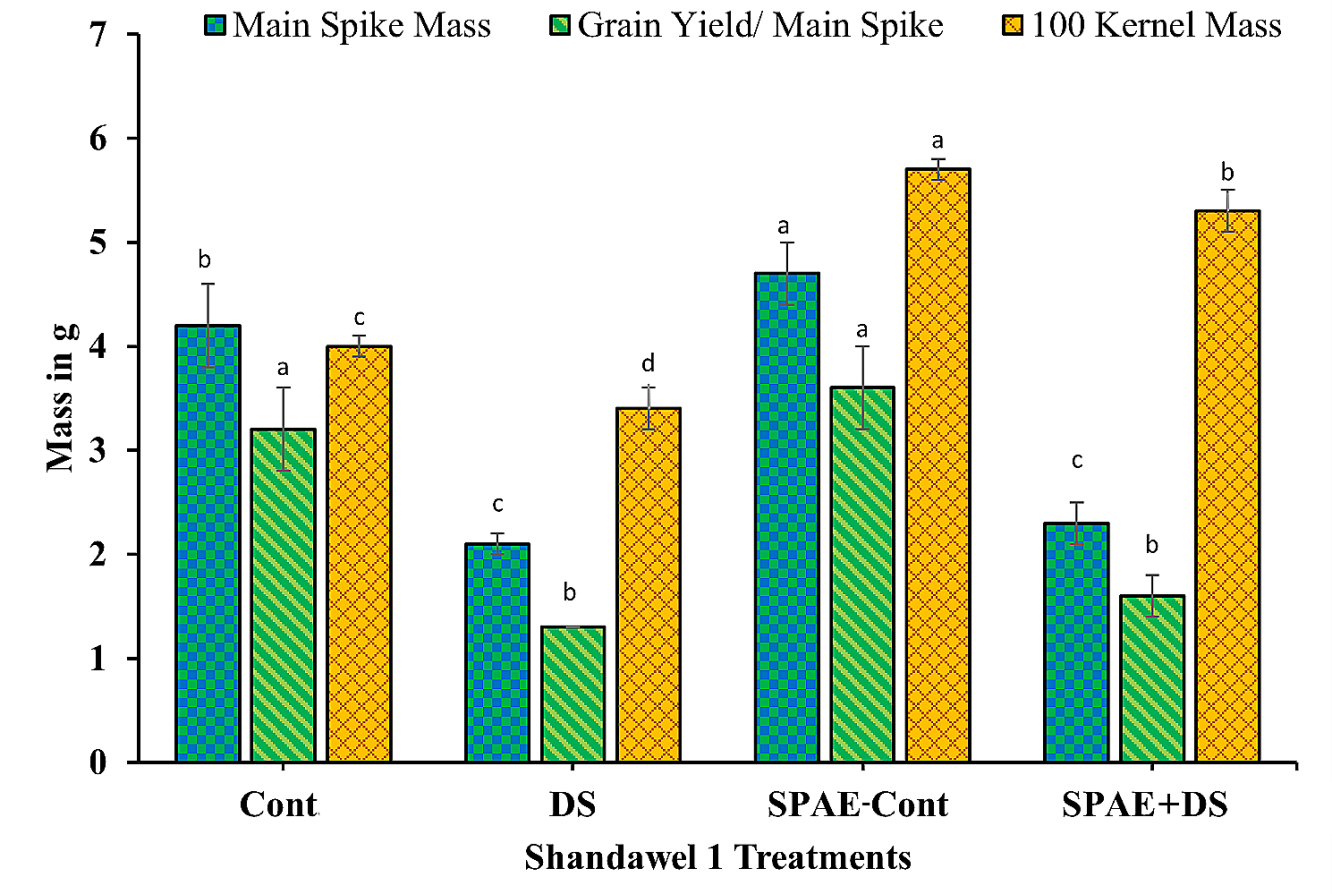
Effect of grain priming in *S. platensis* aqueous extract on main spike mass, grain yield/ main spike and 100 kernel mass of Shandawel 1 cultivar

In the context of sensitive wheat cultivar Shandawel 1 (Fig. 13), the control treatment registered a main spike mass of 4.2 ± 0.4 g, which significantly diminished by 50.0% under DS to 2.1 ± 0.1 g. However, the SPAE-Cont treatment showed a significant main spike mass increment, recording 4.7 ± 0.3 g. The combined SPAE + DS treatment resulted in a notable decrease of 51% compared to the SPAE-Cont treatment but still held a 10% non-significant advantage over DS alone. This reiterates that priming in *S. platensis* might alleviate some adverse impacts of drought stress on the main spike mass. Concerning grain yield per main spike, DS treatment significantly decreased 59% from the control’s 3.2 ± 0.4 g. The SPAE-Cont treatment yielded a non-significant induction (3.6 ± 0.4 g) oner the Cont treatment. However, the combined SPAE + DS treatment resulted in a 56% significant decrease from SPAE-Cont but still had a 23% non-significant advantage over DS alone, demonstrating the potential benefits of *S. platensis* in maintaining grain yield per main spike under drought conditions. Observing 100 kernel mass, DS led to a 15% significant reduction from the control’s 4.0 ± 0.1 g, while the SPAE-Cont displayed an impressive 43% enhancement (5.7 ± 0.1 g). The combined SPAE + DS treatment led to a 7.0% reduction from SPAE-Cont but surpassed DS treatment by 56%. On the other hand, the treatment with SPAE under drought conditions eliminates the effect of the drought reduction and boosts the kernel mass to 5.3 g compared to only 4 g in the control, which is a 33% significant increase.

**Fig. 14:**
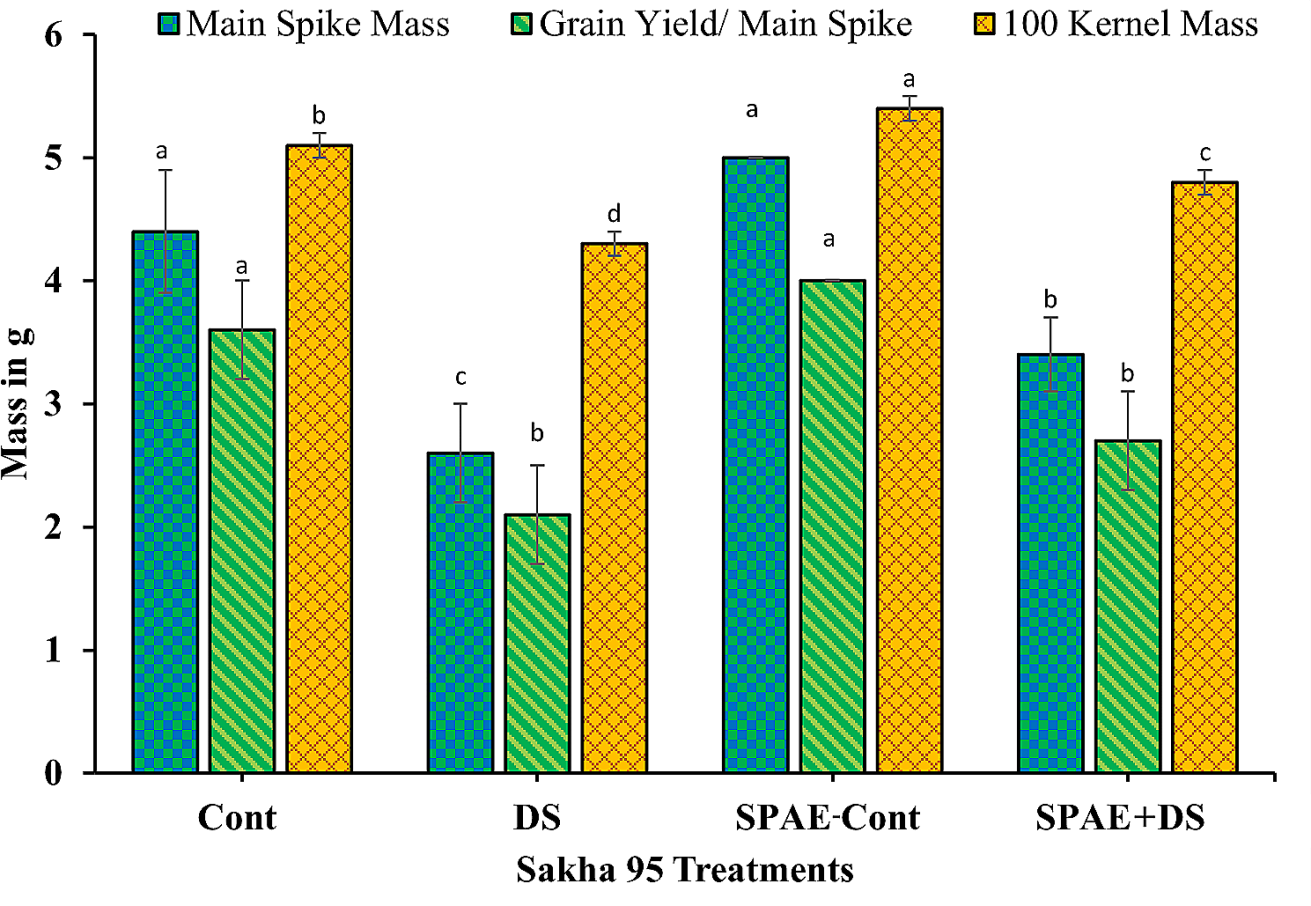
Effect of grain priming in *S. platensis* aqueous extract on main spike mass, grain yield/ main spike and 100 kernel mass of Sakha 95 cultivar

Regarding the tolerant cultivar Sakha 95, it exhibited a different trend (Fig. 14). There was a decline of 41% from 4.4 ± 0.5 g under DS and a trivial 14% increase under SPAE-Cont (5.0 ± 0.0 g) from the control condition for the main spike mass. The combined SPAE + DS treatment witnessed a decrease of 32% from SPAE-Cont, yet it was still 31% higher than DS alone. This further attests to the mitigating role of *S. platensis* against drought stress. Similar patterns were observed for grain yield per main spike and 100 kernel mass in Sakha 95. DS triggered a 42% significant decrease in grain yield per main spike from the control’s 3.6 ± 0.4 g and a minor 10% non-significant induction under SPAE-Cont. Meanwhile, the combined SPAE + DS treatment led to a 33% significant decrease in comparison with SPAE-Cont, but it was still 29% non-significantly higher than DS alone. For 100 kernel mass, DS prompted a 16% significant decrease from the control, while SPAE-Cont showed a 6% significant increase. The combined SPAE + DS treatment resulted in an 11% decrease from SPAE-Cont, but it was still 12% significantly higher than DS alone.

**Fig. 15:**
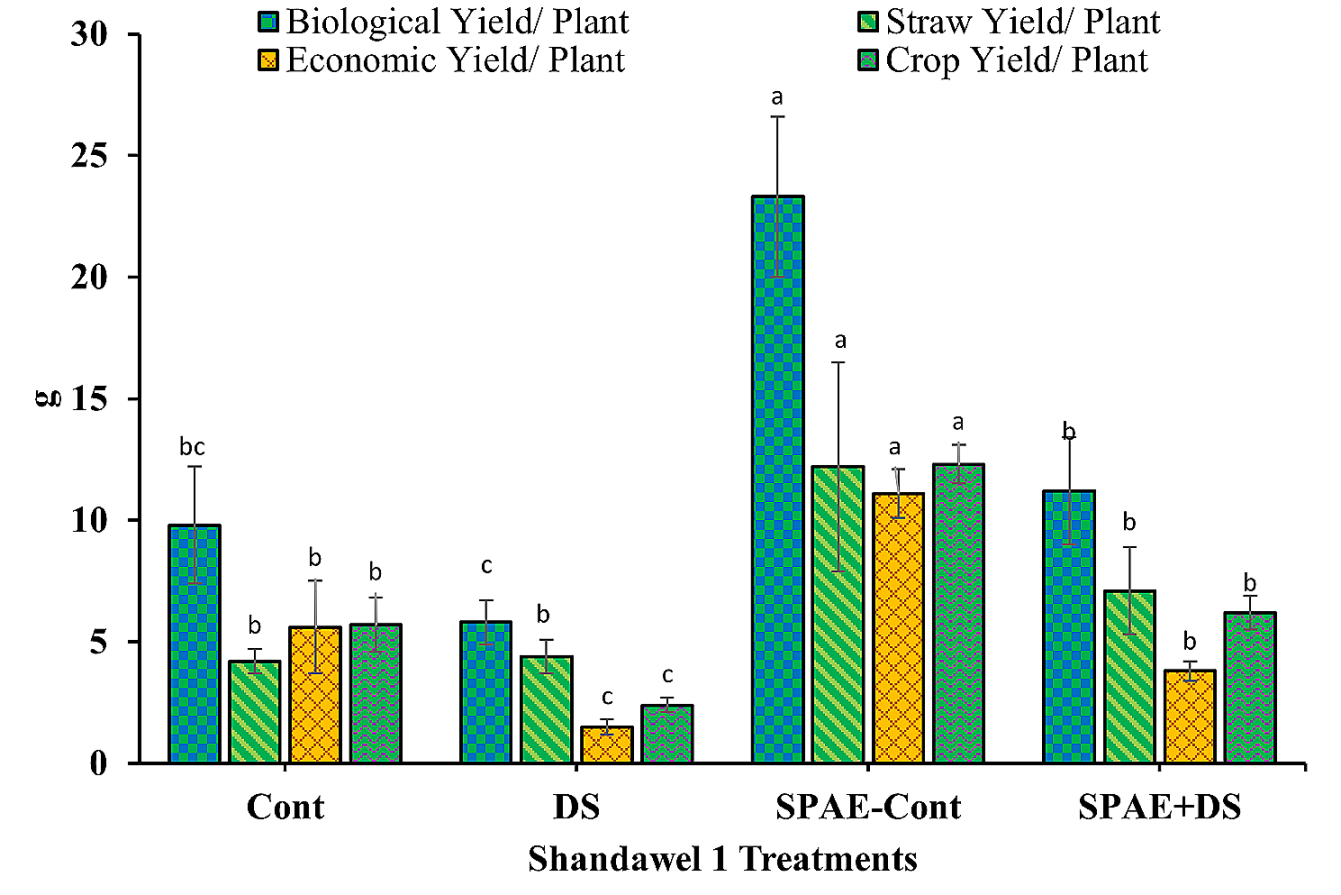
Effect of grain priming in *S. platensis* aqueous extract on biological, straw, economic and crop yield/ plant of Shandawel 1 cultivar

For the sensitive wheat cultivar, Shandawel 1 (Fig. 15), the control treatment resulted in a biological yield per plant equal to 9.8 ± 2.4 g. Under the DS conditions, this yield non-significantly fell by 69% to 5.8 ± 0.9 g. In contrast, the SPAE-Cont treatment demonstrated an impressive increase (138%), resulting in a biological yield of 23.3 ± 3.3 g. When combined with DS (SPAE + DS), the biological yield per plant diminished by 53% compared to SPAE-Cont and maintained a 93% significant increment compared to DS alone, and a 14% non-significant increment compared to Cont treatment. Analogous patterns were seen in the straw yield per plant, with a slight increment under DS from the control’s 4.2 ± 0.5 g, whereas SPAE-Cont resulted in a 190% considerable increase (12.2 ± 4.3 g). In contrast, the SPAE + DS treatment led to a substantial reduction from SPAE-Cont but retained a 61% increase over DS alone and a 69% increase over Cont treatment. Similarly, the economic yield per plant dropped from 5.6 ± 1.9 g under control conditions to 1.5 ± 0.3 g under DS, a significant decrease of 73%. The SPAE-Cont exhibited the highest yield (11.1 ± 1.0 g), highlighting the potential benefits of *S. platensis*. Even under combined SPAE + DS treatment, the economic yield still held a 153% advantage over DS alone. Regarding crop yield per plant, DS caused a 58% reduction from the control’s yield of 5.7 ± 1.1 g. Nevertheless, the crop yield under SPAE-Cont showed an impressive enhancement (12.3 ± 0.8 g). The combined SPAE + DS treatment led to a considerable 50% reduction from SPAE-Cont, but it was still significantly superior (159%) to DS alone.

**Fig. 16:**
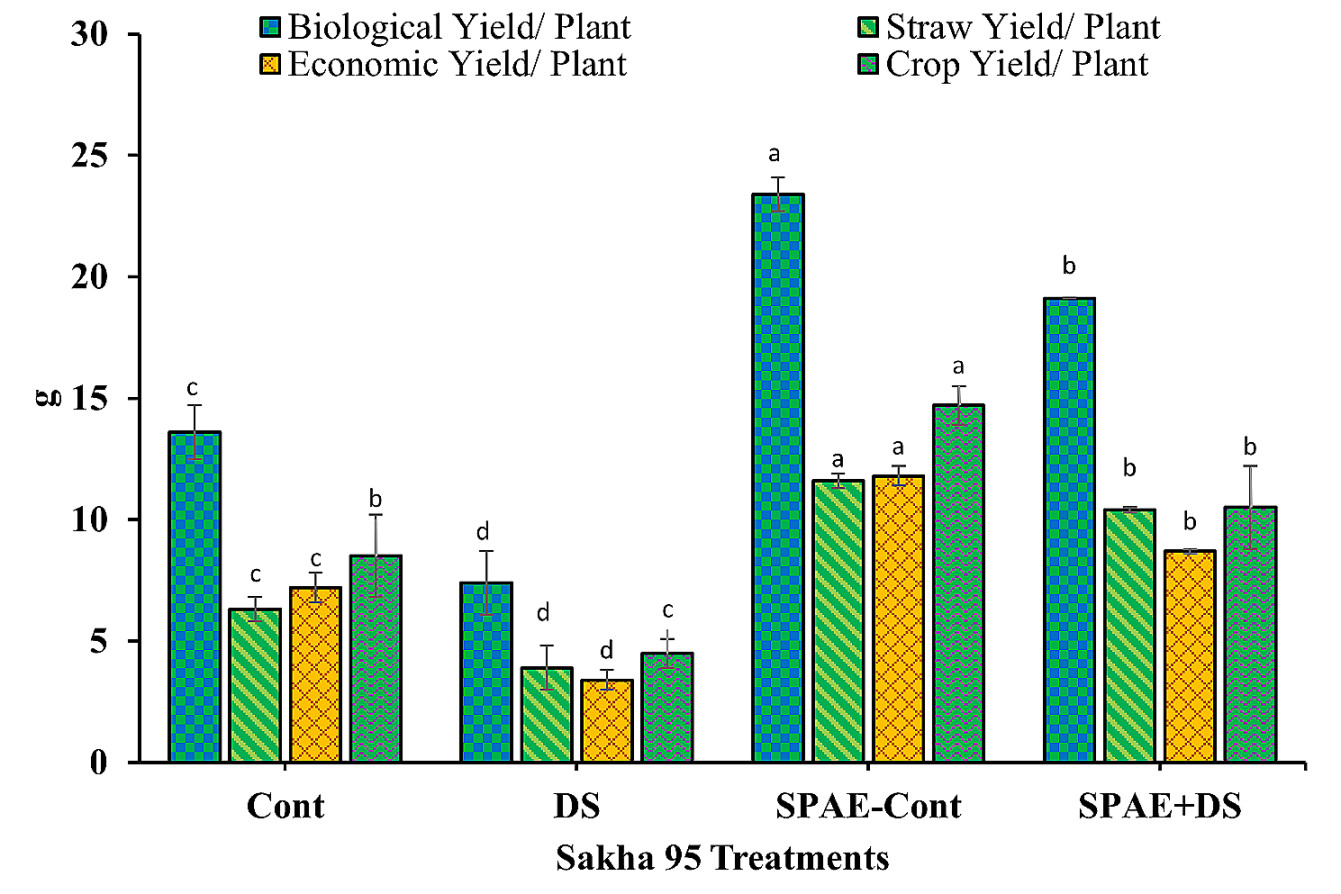
Effect of grain priming in *S. platensis* aqueous extract on biological, straw, economic and crop yield/ plant of Sakha 95 cultivar

Parallel results were observed with the tolerant cultivar, Sakha 95 (Fig. 16). A decrease of 46%, 38%, 53%, and 47% was noted in biological, straw, economic, and crop yields, respectively, under DS compared to control conditions. The beneficial effect of *S. platensis* was again clear in SPAE-Cont conditions, with significant improvements observed in all parameters. The combined SPAE + DS treatment resulted in reductions from the SPAE-Cont. Still, it maintained considerable advantages over DS alone in all yield parameters, again reiterating the potential role of *S. platensis* in mitigating drought stress.

**Fig. 17:**
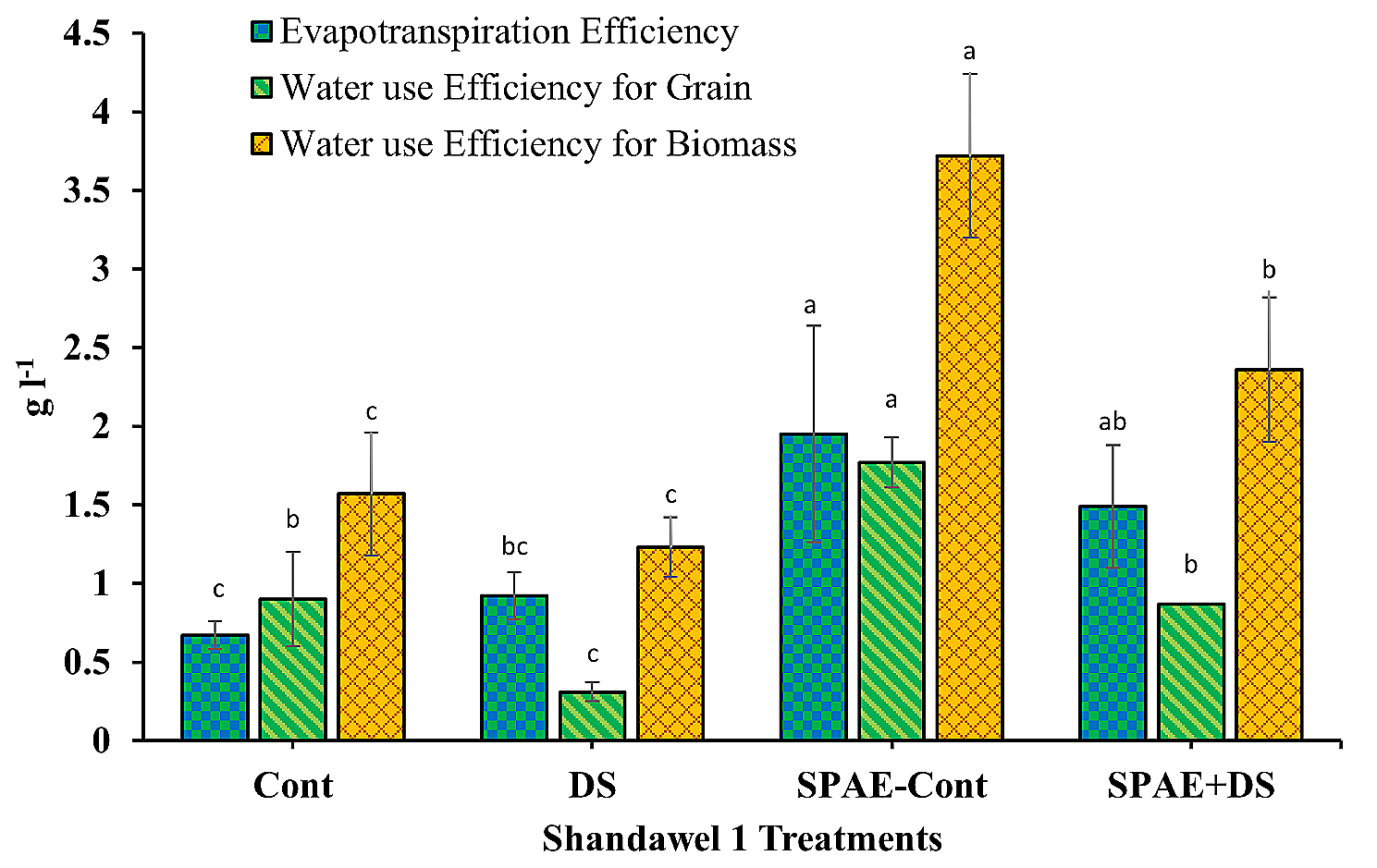
Effect of grain priming in *S. platensis* aqueous extract on evapotranspiration efficiency, water use efficiency for grain and water use efficiency for biomass of Shandawel 1 cultivar

The evapotranspiration efficiency in Shandawel 1 (Fig. 17) was 0.67 ± 0.09 in the Cont treatment. This value escalated non-significantly by 37% under DS to 0.92 ± 0.15. However, the SPAE-Cont treatment exhibited a whopping 191% increment, recording 1.95 ± 0.69. The combined SPAE + DS treatment resulted in a 25% decrease in evapotranspiration efficiency compared to the SPAE-Cont treatment but still non-significantly boasted a 62% edge over DS alone. Hence, pretreatment with *S. platensis* may palliate some detrimental effects of drought stress on evapotranspiration efficiency. For water use efficiency (WUE) for grain, Shandawel 1 displayed a value of 0.90 ± 0.30 under the control treatment plummeting by 66% under DS to 0.31 ± 0.06. Contrastingly, the SPAE-Cont treatment boosted WUE for grain to 1.77 ± 0.16, marking a 97% surge from the control condition. The combined SPAE + DS treatment showed a 3% non-significant reduction compared to the Cont treatment, yet it was 180% significantly higher than DS alone, reaffirming *S. platensis*’s alleviating role against drought stress. Regarding WUE for biomass, under control conditions, Shandawel 1 marked a value of 1.57 ± 0.39, which decreased non-significantly by 21% under DS to 1.23 ± 0.19. SPAE-Cont treatment, however, resulted in a substantial increase of 137% from control, registering 3.72 ± 0.52. The combined SPAE + DS treatment witnessed a 37% decrease compared to SPAE-Cont but still significantly maintained a 91% advantage over DS alone and a 50% advantage over the Cont treatment.

**Fig. 18:**
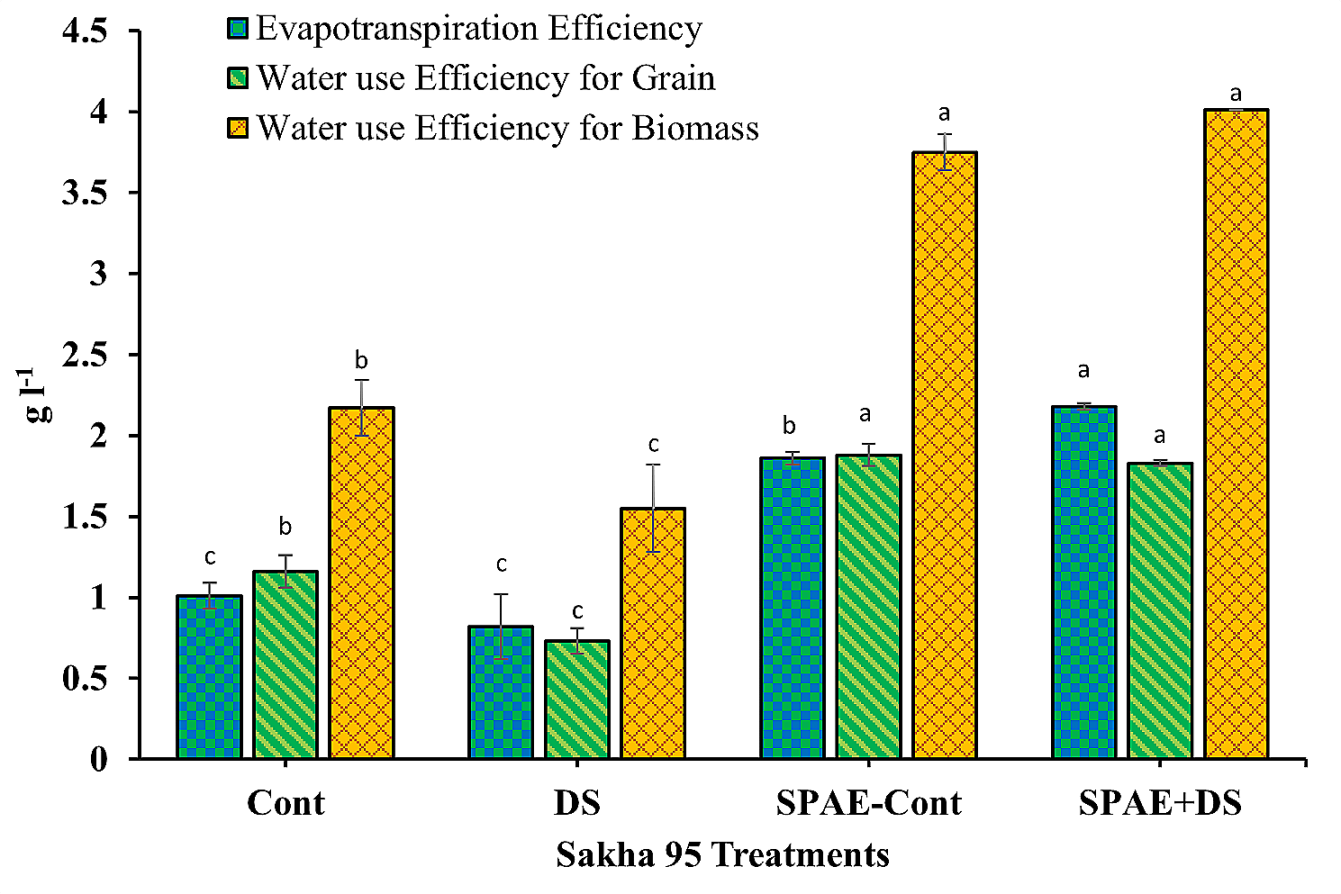
Effect of grain priming in *S. platensis* aqueous extract on evapotranspiration efficiency, water use efficiency for grain, and water use efficiency for biomass of Sakha 95 cultivar

The tolerant cultivar Sakha 95 showed similar trends in all three parameters (Fig. 18), evapotranspiration efficiency, WUE for grain, and WUE for biomass. The relative changes from control conditions to DS, from control to SPAE-Cont, from SPAE-Cont to the combined SPAE + DS, and from SPAE + DS to DS treatment for all these parameters reiterated the potential of *S. platensis* as a drought stress mitigator.

According to 2WCR with LSD test at *p* ≤ 0.05, the interaction between grain priming and watering level factors had a high effect in Shandawel 1 (*** at *p* ≤ 0.05) for peduncle length. In contrast, this interaction varied from moderate too low to non-significant for the other yield attributes as shown in Supplementary Table 3. The interaction had a high effect in Sakha 95 (*** at *p* ≤ 0.05) for the number of spikelets/ main spike only. In contrast, this interaction reveals low significance for plant height, shoot length, and the number of grains/ plant, and non-significance for the rest of the yield parameters as shown in Supplementary Table 4.

### Alterations in biochemical aspects of yielded grains

**Fig. 19:**
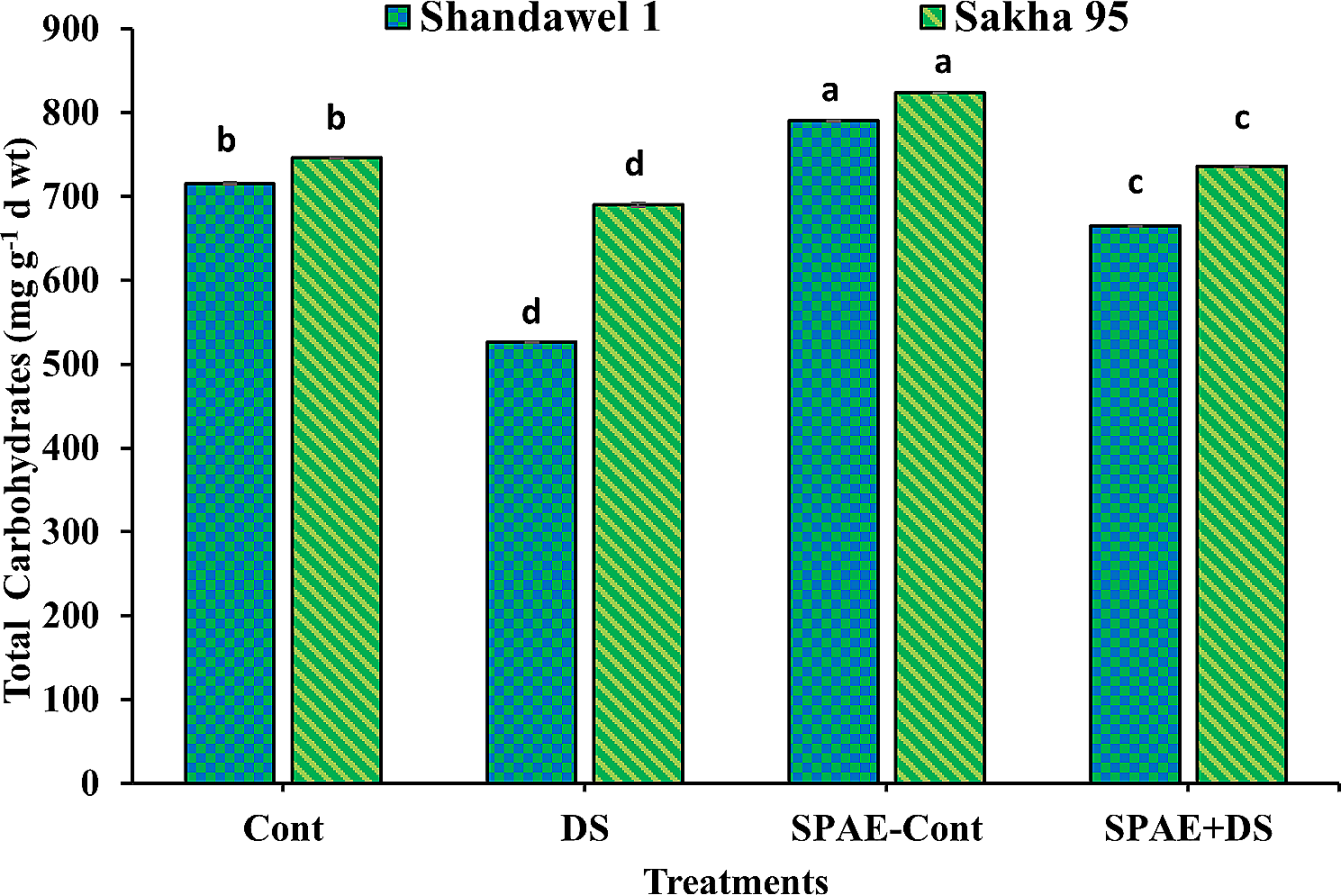
Effect of grain priming in *S. platensis* aqueous extract on the total carbohydrate content of the yielded grains of Shandawel 1 and Sakha 95 cultivars

In Shandawel 1, the control group presented a carbohydrate concentration of 715.5 ± 0.9 mg g^− 1^ d wt as shown in Fig. 19, which significantly declined by 27% under DS to 526.2 ± 0.4 mg g^− 1^ d wt. Conversely, SPAE-Cont treatment enhanced carbohydrate concentration by 10% to 790.0 ± 0.4 mg g^− 1^ d wt, and despite a 16% reduction from SPAE-Cont to SPAE + DS, the latter still marked a 26% increase over DS alone. Similarly, Sakha 95 showed a decline of 7.5% in carbohydrate concentration under DS from the control’s 746.2 ± 0.6 mg g^− 1^ d wt to 690.0 ± 2.8 mg g-1 d wt. SPAE treatments in Sakha 95 also demonstrated an increase in carbohydrate concentration with SPAE-Cont at 823.9 ± 0.4 mg g^− 1^ d wt (10% increase) and SPAE + DS at 7% higher than DS alone despite a 11% decrease from SPAE-Cont.

**Fig. 20:**
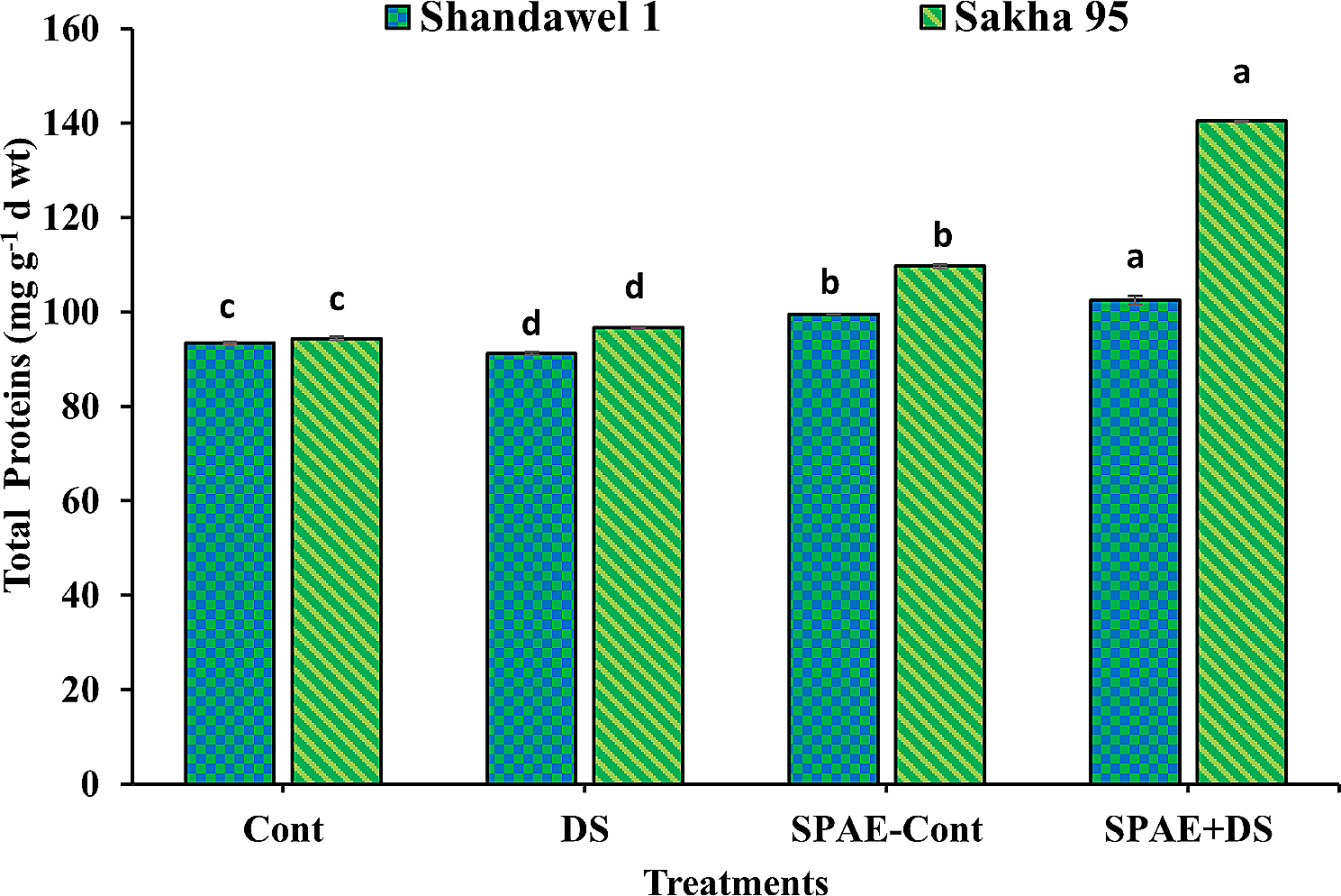
Effect of grain priming in *S. platensis* aqueous extract on the total protein content of the yielded grains of Shandawel 1 and Sakha 95 cultivar

Regarding total protein content, as shown in Fig. 20, Shandawel 1’s control condition showed a protein concentration of 93.4 ± 0.3 mg g^− 1^ d wt, slightly decreasing under DS to 91.2 ± 0.3 mg g^− 1^ d wt. The SPAE-Cont and SPAE + DS treatments increased protein concentrations to 99.6 ± 0.1 and 102.5 ± 0.9 mg g^− 1^ d wt, respectively, indicating significant improvements over control and DS conditions. Sakha 95 exhibited a more pronounced response, with SPAE treatments significantly boosting protein concentrations from the control’s 94.4 ± 0.4 mg g^− 1^ d wt to 109.7 ± 0.4 mg g^− 1^ d wt in SPAE-Cont and a remarkable 140.5 ± 0.2 mg g^− 1^ d wt in SPAE + DS, representing a 52% significant increase over the DS treatment.

According to 2WCR with LSD test at *p* ≤ 0.05 as shown in Supplementary Tables 3 and 4, the interaction between grain priming and drought had a high effect in both cultivars (*** at *p* ≤ 0.05) for grain total carbohydrate content. The interaction had a low effect in Shandawel 1 (* at *p* ≤ 0.05) for grain total protein content but it had a high effect in Sakha 95 (*** at *p* ≤ 0.05) for grain total protein content.

## Discussion

Flag Leaf is the most expanded wheat organ, with its agronomic features being highly affected by drought. The data obtained from the recent study indicated that drought caused a significant decrease in fresh mass, dry mass, water content, succulence degree, succulence quotient, area, and specific area of the flag leaf of the two studied cultivars. The percentage of the decrease in those parameters was higher in the Shandawel 1 cultivar than in the Sakha 95 cultivar, indicating the sensitivity of Shandawel 1 and the tolerance of Sakaha 95. The reduction in those parameters due to drought aligns with findings reported in other drought-related studies [56–58].

The diminution in flag leaf biomass, water content, succulence degree, succulence, succulence quotient, area, and specific area can be ascribed to several drought-induced phenomena: (i) a decline in cellular turgor pressure, ostensibly obstructing cell division, enlargement, and differentiation [59], (ii) an encumbered assimilate supply, largely attributed to the limitations imposed on critical plant processes, predominantly photosynthesis [60], (iii) a perturbation in nutrient availability, concomitant with a diminished water supply [61], and/or, (iv) a temporal shift, manifesting as delayed leaf emergence and precipitated leaf senescence [62].

From a different perspective, it is proposed that the observed reduction in leaf biomass may manifest as an intrinsic adaptive mechanism invoked by the studied wheat cultivars to grapple with water scarcity [63]. Alternatively, the observed decreases in leaf area and specific area might be interpreted as adaptive strategies for drought tolerance, achieved by (i) minimizing the transpiring surface area and conserving water [64] and (ii) safeguarding crucial energy reserves, including carbohydrates [65]. This retardation in leaf growth under drought conditions may be viewed either as a negative impact of water stress or as an adaptation to cope with such challenging environments.

Researchers identified two primary metrics for evaluating leaf succulence: the succulence degree and the succulence quotient [66]. The succulence degree, quantifying water content per unit leaf area, is thought to reflect a plant’s adaptation to arid conditions, with more succulent organs presumably indicating greater water storage capabilities. The succulence quotient, gauging water content per unit of organic matter, is believed to offer insights into the energy expenditure of a leaf for water conservation. Moreover, the degree of leaf sclerophylly is defined as the dry mass accumulated per unit leaf area increased in response to drought conditions based on three hypotheses, positing sclerophylly as (i) an adaptation to water scarcity, (ii) a result of nutrient deficiency, and (iii) a tactic to extend leaf lifespan and boost carbon acquisition [67].

A significant enhancement in flag leaf agronomic features upon the administration of *S. platensis* aqueous extract (SPAE) for both unstressed (SPAE-Cont treatment) and stressed (SPAE + DS) plants was noted in both cultivars. This enhancement is consistent with previous studies showing that *S. platensis* can act as a biostimulant to enhance plant growth [68] and ameliorate stress [69]. The multifaceted benefits of the SPAE, even amidst water stress scenarios, can be ascribed to many intrinsic properties and external interactions. Firstly, nutritional, and bioactive benefits: the SPAE boasts an ensemble of vital nutrients and bioactive compounds. These include but are not limited to, amino acids, vitamins, minerals, and antioxidants, which are reputed for bolstering plant growth and conferring resilience against stresses. Their roles include fortifying photosynthesis, optimizing water dynamics, and buttressing plant health and vitality [70]. Secondly, modulation of plant growth hormones: the hypotheses posit that SPAE potentially acts as a catalyst, stimulating the biosynthesis of crucial plant growth hormones like auxins and cytokinins. These hormones are cardinal regulators of plant physiological dynamics, encompassing cellular division, differentiation, and extension. Such modulation can manifest as heightened biomass production, refined root architecture, and an uptick in nutrient assimilation efficiency [71]. Thirdly, alleviation of oxidative stress: the antioxidative attributes of SPAE are crucial, especially under water-stressed scenarios. By limiting oxidative stress in plant tissues, the extract appears to offset the deleterious impacts of water stress, culminating in enhanced plant growth and function [72].

Drought notably impacts photosynthesis, and the pigmentation system can directly indicate its efficiency. Therefore, determining the amount of photosynthetic pigment in wheat under drought conditions was of paramount importance for this study. The current study revealed that drought led to a notable decrease in chlorophyll-a, chlorophyll-b, and total chlorophyll content of flag leaves in both cultivars. The reduction in the content of chlorophyll aligns with findings from similar research on stressed wheat and stress-affected plants [73, 74]. Several factors could account for this reduction: pigment breakdown from accumulated reactive oxygen species [75], diminished pigment synthesis [76], increased activity of chlorophyllase breaking down pigments [77], or disruptions in the synthesis of proteins crucial to chlorophyll [78]. Furthermore, the reduced presence or the plant’s diminished capacity to absorb elements like Mg and Fe can decrease pigment content.

The results also showed a substantial increase in the content of carotenoids due to the drought conditions, comparable to the increase observed in a study on wheat subjected to drought stress [79]. It is found that, determined that carotenoids are crucial under stress conditions, fulfilling several key functions: (i) capturing light via singlet state energy transfer, (ii) maintaining plastid structure integrity, (iii) safeguarding chlorophylls by quenching their triplet state, (iv) neutralizing certain reactive oxygen species (ROS), particularly singlet oxygen, and (v) dissipating surplus energy [80]. The increased ratio of carotenoids to total chlorophylls due to drought might be attributed to the crucial role that carotenoids play in various protective mechanisms [81]. When examining the CSI, an indicator of pigment stability under stress, higher values hint at a greater chlorophyll availability, aiding plants in combating stress by enhancing their photosynthetic output [82]. The current study indicated that drought led to a significant drop in CSI, as observed in another study on wheat [83].

*S. platensis* treatments reinforced the pigmentation in both stressed and unstressed wheat. Multiple reasons could explain the enhanced pigmentation in wheat treated with SPAE. It is plausible that the extract, rich in pigments like phycocyanin, chlorophyll, and carotenoids, could be absorbed by the plants, thus amplifying their pigmentation system and overall resilience [84]. Moreover, the extract might activate genes responsible for pigment synthesis in plants, as previous work has shown its effects on tomatoes and rice [85]. The results resonate with other recent studies that highlight the positive effects of biostimulants in mitigating plant stress [86].

Foliage gas exchange characteristics are pivotal in influencing plant photosynthetic performance due to genetic and environmental variances [87]. The photosynthesis rate, a key driver of plant productivity, has been notably identified as a chief indicator predominantly impacted by drought conditions. Observations from the current research revealed a significant decline in the photosynthetic rate due to drought in both cultivars, matching the results obtained from other studies on different plants [88, 89]. The photosynthesis rate often declines because of drought due to either stomatal or non-stomatal factors. Stomatal limitations are commonly associated with reduced CO_2_ availability as stomata tend to close. While this stomatal closure primarily serves as a protective response against tissue dehydration, it may restrict CO_2_ availability or assimilation [90]. On the other hand, non-stomatal limitations can arise from (i) limited CO_2_ diffusion through the mesophyll, (ii) compromised activity of crucial enzymes, notably ribulose-1,5-bisphosphate carboxylase/oxygenase (RuBisCO), (iii) degradation of leaf cellular architecture, or (iv) decreased CO_2_ permeability due to dehydration’s adverse effects on leaf cell walls, plasma membranes, and cuticle [91].

The study’s findings reveal that SPAE significantly boosts the photosynthetic rate in two wheat cultivars under SPAE-Cont compared to the Cont treatment, suggesting that *S. platensis* enhances photosynthetic processes. Moreover, under SPAE + DS, photosynthesis rates exceeded those observed under DS alone, highlighting *S. platensis’s* protective effects on photosynthetic machinery against the adverse impacts of drought. This enhancement may be caused by antioxidants in *S. platensis*, like phycocyanin. This pigment-protein complex collaboratively functions with chlorophyll in the process of photosynthesis, which is shown to scavenge free radicals, safeguard cells against oxidative damage, and maintain photosynthetic efficiency [92]. Likewise, plant growth stimulators in *S. platensis*, such as auxins and cytokinins [93], potentially boost growth and photosynthesis under stress. Prior work supports SPAE-enhanced photosynthesis under stress [94].

In the study findings, the transpiration rate in the two wheat cultivars declined significantly due to the drought. The reduced rates of transpiration under DS and SPAE + DS treatments are consistent with findings from other research involving different plants [95, 96]. It’s theorized that this drop in transpiration rate, attributed to water shortages, is due to an inhibition of photosynthesis, which, coupled with CO_2_ accumulation in the stomatal guard cells, may lead to partial or complete stomatal closure [97]. The identified decrease in transpiration rate may be viewed as an adaptive mechanism for the wheat plants to endure drought conditions.

Additionally, the process by which a plant leaf achieves carbon gain via photosynthesis in relation to water loss through transpiration has been identified in this study as pWUE. The findings of the present investigation highlight that drought conditions led to a notable reduction in the pWUE of Shandawel 1, whereas Sakha 95 experienced a negligible decrease which aligns with studies that reported a downturn in pWUE for certain wheat strains under drought conditions [45]. This reduction in pWUE under drought circumstances might stem from the adverse impacts of stress on the rate of photosynthesis and transpiration. However, wheat cultivars like Sakha 95, which exhibit minimal changes in pWUE during stress, might suggest an ability to maintain substantial or at least decent biomass build-up without significant water loss [48].

This study’s results demonstrate SPAE’s potential to significantly enhance pWUE, matching with the study that reported increased pWUE in plants treated with a biostimulant [98]. This pWUE enhancement could be attributed to; (i) SPAE is rich in bioactive compounds reported to have antioxidant properties which help in protecting the plant cells from oxidative damage caused by drought stress, thus maintaining cellular function and potentially allowing for more efficient water use, (ii) applying SPAE could potentially influence stomatal behaviour, leading to a more controlled water loss and enhanced carbon dioxide uptake under drought conditions [99].

Stomatal conductance (gs) is a critical parameter in plant physiology, reflecting the ability of stomata to regulate the exchange of gases between the leaf and the atmosphere [100]. It is a key factor influencing CO_2_ uptake by plants [101]. It is intimately linked with photosynthetic and water use efficiency, especially under drought-stress conditions [102]. In this study, the sensitive wheat cultivar Shandawel 1 showed a substantial decrease in gs under DS. It is a typical response to drought, as plants tend to close their stomata to conserve water, which can reduce photosynthetic capacity [103]. Applying SPAE-Cont under non-stress conditions led to a significant increase in gs, which suggests that SPAE may stimulate stomatal opening or improve leaf water status, as has been observed in other studies where biostimulants increased gs [104]. When Shandawel 1 was treated with SPAE + DS, gs significantly improved compared to DS treatment alone. It indicates that SPAE, as its biostimulant effect, may provide some mitigation of drought-induced stomatal closure, potentially due to the presence of osmoprotectants, hormones, or other stress-alleviating compounds in the extract [105].

The drought-tolerant cultivar Sakha 95 had a higher baseline gs under control conditions, reflecting its greater tolerance to water stress. It is consistent with previous research indicating that drought-tolerant cultivars often maintain higher gs under stress conditions [106]. Interestingly, the SPAE-Cont treatment for Sakha 95 led to a decrease in gs, which is lower than the control but still higher than the SPAE + DS treatment, further reducing gs. Mesophyll conductance (gm) is an essential factor influencing CO_2_ diffusion within the leaf [107]. The results of this study show a significant enhancement in gm in both cultivars following SPAE treatment and SPAE + DS treatment when compared to DS treatment only. It suggests that *S. platensis* pre-treatment mitigates the adverse impacts of drought stress on mesophyll conductance. For instance, a study demonstrated increased gm in plants treated with biostimulants under drought stress [108]. Increasing gm with SPAE treatment suggests improved internal CO_2_ diffusion, which could enhance photosynthetic efficiency [109]. The higher gm in SPAE-Cont treatments could be indicative of alterations in leaf anatomy or biochemistry, such as increased chloroplast distribution or aquaporin activity [110].

Carbohydrates, the primary photosynthetic products, are one of the most important organic components of the plant’s cellular dry matter; their amounts fluctuate highly under drought stress [111]. Various experimental studies could document the accumulation of carbohydrates in different plants because of limited water supply [112, 113]. Accumulating carbohydrates under stressful conditions can be considered a potent tolerance strategy, could decrease cellular water potential, and contribute to the avoidance of ROS-induced oxidative injury. As another carbohydrate, trehalose was generally reported to be present in relatively trace amounts under control conditions, while its concentration significantly increased on plant exposure to stress. In this context, it was assumed that trehalose accumulation in plants facing drought is an effective strategy to cope with stress since trehalose could conserve the photosynthetic electron transport chain, stabilize proteins and membrane lipids, quench free radicals, and sustain osmotic adjustment [114]. Drought-induced reduction in leaf polysaccharide content in Shandawel 1 can be attributed to the stress caused by a drop in leaf chlorophyll content with consequent suppression of photosynthetic efficiency and carbon gain. Alternatively, such a drop in leaf polysaccharide content could be considered a trial from the stressed plants to withstand stress by obtaining simpler sugars from polysaccharides to sustain proper growth [115].

The findings of this study demonstrate that the application of *S. platensis* significantly enhances the concentration of TSS in both cultivars, particularly under drought-stress conditions. These results are consistent with previous research highlighting the potential of *S. platensis* as a biofertilizer in enhancing the growth of *Lupinus luteus* by an accumulation of soluble sugars [116]. The significant increase in TSS content following SPAE + DS treatment suggests that *S. platensis* may enhance the overall soluble sugar content of the wheat cultivars, which is a crucial factor in plant stress tolerance. Soluble sugars play a vital role in osmotic adjustment, scavengers, and signalling under stress conditions [117]. Furthermore, the more significant increase in trehalose, polysaccharides, and total carbohydrate content under the SPAE + DS treatment suggests that *S. platensis* may synergistically affect drought stress in enhancing sugar metabolism. This increase is in line with the findings of another study that reported that biostimulants can enhance plant stress responses by modulating metabolic pathways and activating stress-related genes [118].

Yield, the paramount economic characteristic of wheat, and its grain production serve as the primary criteria for drought tolerance. In this study, a significant reduction was observed in yield attributes due to water stress in both wheat cultivars, with Sakha 95 experiencing a lesser decrease, matching with other studies [119, 120]. The decrease in wheat yield due to water stress can be linked to drought’s inhibitory impact on plant growth, stemming from the suppression of various metabolic processes. Additionally, drought conditions necessitate high energy and carbohydrate expenditure for osmoregulation and disrupt normal cell functions, further contributing to the reduction in photosynthesis and overall yield [121].

The study provides compelling evidence that the application of SPAE wheat grains in SPAE can greatly enhance yield productivity compared to the control treatment and also mitigate the adverse impacts of drought on yield attributes compared to the drought stress treatment in both cultivars. This enhancement effect, rooted in the extract’s nutritional, growth-promoting, and stress-mitigating properties, resonates with the findings of recent literature demonstrating the potential of algal biostimulants to improve crop resilience under abiotic stresses [72]. The positive effect of SPAE on wheat height, shoot length, spike length, and tiller number under drought stress corroborates earlier work where an algal extract increased tillering and maintained plant height in water-stressed wheat and these effects to enhanced nutrient absorption and growth hormone levels stimulated by the biostimulants [122].

The increase in spike number, spikelets number, and grains number per spike and plant due to SPAE aligns with another study that reported improved spike characteristics and grain number in droughted wheat treated with seaweed extracts compared to untreated controls [123]. This increase reflects the potential of algal biostimulants to ensure efficient flowering and grain development even under moisture-deficit conditions. The study observation that SPAE maintained economic grain yield under drought parallels results in a study [124], where brown algal extracts significantly improved grain yield in drought-stressed wheat by increasing assimilation and remobilization of stem reserves to grain. The positive impact of SPAE on yield attributes was more explicit in the drought-sensitive Shandawel 1 cultivar than in the tolerant Sakha 95, in agreement with other study, wheat cultivars differing in drought susceptibility responded differently to biostimulant application under water stress [125].

Drought stress has been recognized as a substantial challenge to crop production and quality, affecting numerous physiological and biochemical parameters in plants [126]. The results showed a decline in carbohydrate concentrations under drought stress conditions in both cultivars. Previous studies have suggested drought stress can hinder plant carbohydrate metabolism, reducing grain quality and yield [127]. The observed reduction could be attributed to reduced photosynthesis and alterations in carbohydrate metabolism induced by drought stress [128]. Interestingly, the application of SPAE appeared to play a pivotal role in offsetting the negative impacts of drought stress, particularly on the carbohydrate content of the sensitive cultivar. The SPAE-Cont enhanced carbohydrate concentrations in both cultivars. Furthermore, when combined with SPAE + DS, the carbohydrate concentration, although decreased compared to the SPAE-Cont, was still higher than DS alone for both cultivars. It suggests that bioactive compounds in *S. platensis* might enhance carbohydrate metabolism or protect against drought-induced oxidative stress [129].

Regarding total protein concentration, drought stress caused a slight reduction in both cultivars. Applying SPAE led to an increase in protein concentration, even surpassing the control values. Remarkably, in the tolerant cultivar, Sakha 95, the combined SPAE + DS treatment showcased a protein concentration of 49.0% higher than DS alone and 28.0% over the control. This marked increase might imply that *S. platensis* compounds not only counteract the adverse effects of drought stress but may also have a synergistic effect, stimulating protein synthesis or accumulation. Some studies have reported that cyanobacterial extracts can enhance nitrogen assimilation, increasing plant protein concentrations [130].

## Conclusion

Severe environmental conditions, particularly drought, significantly inhibit agricultural development by challenging plant growth and productivity. Drought stress and diminishing water resources impair these challenges, necessitating innovative agriculture strategies to boost cereal production in the face of rising global food demand. Water’s role in plant growth underscores the necessity for understanding and improving plants’ drought resistance mechanisms. This research has highlighted the complex adaptive responses of wheat to drought, revealing the promising role of *Spirulina platensis* aqueous extract (SPAE) in enhancing plant resistance. SPAE-treated wheat exhibited improved agronomic performance, physiological attributes, and biochemical profiles, even under drought conditions, signifying that the extract could be used as a biostimulant. The study findings contribute to understanding plant responses to drought and the beneficial application of biostimulants, offering valuable insights for sustainable agricultural practices and food security.

### Electronic supplementary material

Below is the link to the electronic supplementary material.

Supplementary Material 1

## Data Availability

No datasets were generated or analysed during the current study.
