# Supplementary material for: Mitigating drought stress in wheat plants (Triticum Aestivum L.) through grain priming in aqueous extract of spirulina platensis
Source: BMC Plant Biol. 2024 Apr 2;24:233. doi: 10.1186/s12870-024-04905-z (PMC10986097; doi:10.1186/s12870-024-04905-z)
Supplement: Supplementary file 1 — Supplementary Material 1 [file 12870_2024_4905_MOESM1_ESM.docx]

Supplementary Table 1 Single effect of each grain priming and watering level on 49 morpho-physiological, and biochemical parameters of Shandawel 1 cultivar.

| parameters | Grain priming | | | Watering Level | | | parameters | Grain priming | | | Watering Level | | |
| --- | --- | --- | --- | --- | --- | --- | --- | --- | --- | --- | --- | --- | --- |
|  | Dist. H_2_O | SPAE | *P*-  value | NI | D | *P* -value |  | Dist.  H_2_O | SPAE | *P*- value | NI | D | *P*- value |
| Leaf Fresh mass (g) | 0.536^b^ | 0.643^a^ | *** | 0.676^a^ | 0.503^b^ | *** | Trehalose (mg g^-1^) | 43.7^b^ | 55.7^a^ | *** | 48.6^b^ | 50.8^a^ | *** |
| Leaf Dry Mass (g) | 0.160^a^ | 0.161^a^ | ns | 0.166^a^ | 0.154^b^ | *** | Polysaccharides (mg g^-1^) | 39.9^b^ | 44.1^a^ | *** | 41.6^b^ | 42.4^a^ | ** |
| Succulence Degree (mg cm^-2^) | 14.2^a^ | 14.6^a^ | ns | 15.7^a^ | 13.1^b^ | *** | Leaf TC (mg g^-1^) | 89.7^b^ | 106.8^a^ | *** | 96.0^b^ | 100.5^a^ | *** |
| Sclerophylly Degree (mg cm^-2^) | 6.2^a^ | 4.9^b^ | *** | 5.1^b^ | 6.0^a^ | *** | Plant Height (cm) | 66.2^a^ | 67.7^a^ | ns | 75.6^a^ | 58.3^b^ | *** |
| Leaf Water Content (g g^-1^) | 0.694^b^ | 0.748^a^ | *** | 0.753^a^ | 0.688^b^ | *** | Shoot Length (cm) | 84.3^a^ | 84.9^a^ | ns | 94.0^a^ | 75.1^b^ | *** |
| Succulence Quotient (mg mg^-1^) | 2.3^b^ | 3.0^a^ | *** | 3.1^a^ | 2.3^b^ | *** | Spike Length (cm) | 17.4^a^ | 17.8^a^ | ns | 19.0^a^ | 16.2^b^ | ** |
| Leaf area (cm^2^) | 26.1^b^ | 32.9^a^ | *** | 32.5^a^ | 26.5^b^ | *** | Peduncle Length (cm) | 12.7^a^ | 13.5^a^ | ns | 16.2^a^ | 10.0^b^ | *** |
| Leaf Specific Area (cm^2^ g^-1^) | 162.4^b^ | 204^a^ | *** | 195^a^ | 171^b^ | *** | No. of Tillers/ Plant | 2.0^b^ | 4.2^a^ | *** | 3.2^a^ | 3.0a | ns |
| Chlorophyll-a (mg g^-1^) | 0.248^b^ | 0.517^a^ | *** | 0.525^a^ | 0.239^b^ | *** | No. of Grains/ Main spike | 44.6^b^ | 50.8^a^ | * | 71.0^a^ | 24.5^b^ | *** |
| Chlorophyll-b (mg g^-1^) | 0.078^b^ | 0.172^a^ | *** | 0.175^a^ | 0.076^b^ | *** | No. of Grains/ Plant | 67.0^b^ | 149.5^a^ | *** | 154.5^a^ | 62.0^b^ | *** |
| Carotenoids (mg g^-1^) | 0.119^b^ | 0.131^a^ | ** | 0.117^b^ | 0.132^a^ | *** | No. of Spikes/ Plant | 1.8^b^ | 3.5^a^ | *** | 3.0^a^ | 2.3^b^ | ** |
| Total Chlorophylls (mg g^-1^) | 0.326^b^ | 0.689^a^ | *** | 0.700^a^ | 0.315^b^ | *** | No. of Spikelets/ Main spike | 19.0^b^ | 22.0^a^ | * | 21.7^a^ | 19.3^b^ | * |
| Chlorophyll-a/ b | 3.083^a^ | 3.083^a^ | ns | 3.084^a^ | 3.082^a^ | ns | No. of Spikelets/ plant | 34.0^b^ | 75.7^a^ | *** | 65.2^a^ | 44.5^b^ | *** |
| Carotenoids/ Total Chlorophylls | 0.470^a^ | 0.222^b^ | *** | 0.185^b^ | 0.506^a^ | *** | Main Spike Mass (g) | 3.1^b^ | 3.5^a^ | * | 4.5^a^ | 2.2^b^ | *** |
| Chlorophyll Stability Index % | 70^a^ | 74^a^ | ns | 100^a^ | 44^b^ | *** | Grain Yield/ Main Spike (g) | 2.3^a^ | 2.6^a^ | ns | 3.4^a^ | 1.5^b^ | *** |
| A (µmol m^-2^ sec^-1^) | 5.4^b^ | 9.0^a^ | *** | 11.2^a^ | 3.2^b^ | *** | 100 kernel Mass (g) | 3.7^b^ | 5.5^a^ | *** | 4.9^a^ | 4.4^b^ | *** |
| E (mmol m^-2^ sec^-1^) | 1.2^a^ | 1.4^a^ | ns | 1.9^a^ | 0.8^b^ | *** | Biological Yield/ Plant (g) | 7.8^b^ | 17.2^a^ | *** | 16.5^a^ | 8.5^b^ | *** |
| pWUE (µmol mmol^-1^) | 4.1^b^ | 6.0^a^ | *** | 6.0^a^ | 4.0^b^ | *** | Straw Yield/ Plant (g) | 4.3^b^ | 9.6^a^ | ** | 8.2^a^ | 5.7^a^ | ns |
| Ls | 0.27^b^ | 0.30^a^ | ** | 0.33^a^ | 0.24^b^ | *** | Economic Yield/ plant (g) | 3.6^b^ | 7.6^a^ | *** | 8.3^a^ | 2.8^b^ | *** |
| gs (mmol m^-2^ sec^-1^) | 61^b^ | 80^a^ | *** | 101^a^ | 40^b^ | *** | Crop yield/ Plant (g) | 4.0^b^ | 9.3^a^ | *** | 9.0^a^ | 4.3^b^ | *** |
| gm (µmol mol^-1^) | 0.021^b^ | 0.037^a^ | *** | 0.047^a^ | 0.012^b^ | *** | Evapotranspiration Eff. | 0.79^b^ | 1.72^a^ | ** | 1.31^a^ | 1.20^a^ | ns |
| Ci (µmol mol^-1^ | 265^a^ | 251^b^ | ** | 241^b^ | 275^a^ | *** | WUE for Grain (g l^-1^) | 0.61^b^ | 1.32^a^ | *** | 1.33^a^ | 0.59^b^ | *** |
| Ci/ gs (µmol mmol^-1^ m^2^ sec) | 5.7^a^ | 4.0^b^ | ** | 2.5^b^ | 7.2^a^ | *** | WUE for Biomass (g l^-1^) | 1.40^b^ | 3.04^a^ | *** | 2.64^a^ | 1.80^b^ | ** |
| TSS (mg g^-1^) | 6.14^b^ | 7.03^a^ | *** | 5.80^b^ | 7.36^a^ | *** | grain TC (mg g^-1^ d wt) | 620.8^b^ | 727.5^a^ | *** | 752.7^a^ | 595.6^b^ | *** |
| ---------------------------- | ----- | ----- | ----- | ----- | ----- | ----- | grain TP (mg g^-1^ d wt) | 92.3^b^ | 101.0^a^ | *** | 95.4^b^ | 97.9^a^ | *** |

The data were represented as mean values, different superscript alphabetical letters refer to significant variation with the least significant difference (LSD) at *p* ≤ 0.05. The low, moderate, and high degrees of significance are indicated by *, **, and ***, while the non-significant difference is abbreviated as ns. Abbreviations: Dist. H_2_O (Distilled water), SPAE (*Spirulina platensis* aqueous extract), NI (normal irrigation), D (drought), A (photosynthesis rate), E (transpiration rate), pWUE (photosynthetic water use efficiency), Ls (stomatal limitation), gs (stomatal conductance), gm (mesophyll conductance), Ci (intercellular CO₂ concentration), TSS (total soluble sugars), TC (total carbohydrate content), Eff. (efficiency), and WUE (water use efficiency).

Supplementary Table 2 Single effect of each grain priming and watering level on 49 morpho-physiological, and biochemical parameters of Sakha 95 cultivar.

| parameters | Grain priming | | | Watering Level | | | parameters | Grain priming | | | Watering Level | | |
| --- | --- | --- | --- | --- | --- | --- | --- | --- | --- | --- | --- | --- | --- |
|  | Dist. H_2_O | SPAE | *P*-  value | NI | D | *P* -value |  | Dist.  H_2_O | SPAE | *P*- value | NI | D | *P*- value |
| Leaf Fresh mass (g) | 0.550^b^ | 0.725^a^ | *** | 0.691^a^ | 0.584^b^ | *** | Trehalose (mg g^-1^) | 59.0^b^ | 66.1^a^ | *** | 57.5^b^ | 67.6^a^ | *** |
| Leaf Dry Mass (g) | 0.165^b^ | 0.200^a^ | *** | 0.185^a^ | 0.180^a^ | ns | Polysaccharides (mg g^-1^) | 44.3^b^ | 47.2^a^ | *** | 43.5^b^ | 48.0^a^ | *** |
| Succulence Degree (mg cm^-2^) | 13.3^b^ | 15.2^a^ | ** | 15.0^a^ | 13.5^b^ | ** | Leaf TC (mg g^-1^) | 110.3^b^ | 121.4^a^ | *** | 107.9^b^ | 123.8^a^ | *** |
| Sclerophylly Degree (mg cm^-2^) | 5.8^a^ | 5.8^a^ | ns | 5.5^b^ | 6.1^a^ | ** | Plant Height (cm) | 77.5^a^ | 80.9^a^ | ns | 85.2^a^ | 73.2^b^ | *** |
| Leaf Water Content (g g^-1^) | 0.695^b^ | 0.724^a^ | ** | 0.732^a^ | 0.687^b^ | *** | Shoot Length (cm) | 93.4^a^ | 96.8^a^ | ns | 102.8^a^ | 87.4^b^ | *** |
| Succulence Quotient (mg mg^-1^) | 2.3^b^ | 2.6^a^ | ** | 2.7^a^ | 2.2^b^ | *** | Spike Length (cm) | 15.0^a^ | 16.1^a^ | ns | 16.2^a^ | 15.0^a^ | ns |
| Leaf area (cm^2^) | 28.7^b^ | 34.6^a^ | *** | 33.8^a^ | 29.5^b^ | *** | Peduncle Length (cm) | 15.1^a^ | 13.2^a^ | ns | 13.9^a^ | 14.3^a^ | ns |
| Leaf Specific Area (cm^2^ g^-1^) | 173.4^a^ | 173.7^a^ | ns | 183.1^a^ | 164.0^b^ | ** | No. of Tillers/ Plant | 3.0^b^ | 4.5^a^ | *** | 3.8^a^ | 3.7^a^ | ns |
| Chlorophyll-a (mg g^-1^) | 0.415^b^ | 0.758^a^ | *** | 0.764^a^ | 0.409^b^ | *** | No. of Grains/ Main spike | 53.0^b^ | 67.3^a^ | ** | 68.3^a^ | 52.0^b^ | ** |
| Chlorophyll-b (mg g^-1^) | 0.144^b^ | 0.255^a^ | *** | 0.262^a^ | 0.137^b^ | *** | No. of Grains/ Plant | 106.7^b^ | 235.0^a^ | *** | 187.0^a^ | 154.7^b^ | ** |
| Carotenoids (mg g^-1^) | 0.188^b^ | 0.199^a^ | ** | 0.183^b^ | 0.203^a^ | *** | No. of Spikes/ Plant | 2.5^b^ | 4.3^a^ | *** | 3.7^a^ | 3.2a | ns |
| Total Chlorophylls (mg g^-1^) | 0.559^b^ | 1.012^a^ | *** | 1.026^a^ | 0.545^b^ | *** | No. of Spikelets/ Main spike | 20.2^b^ | 22.0^a^ | *** | 22.0^a^ | 20.2^b^ | *** |
| Chlorophyll-a/ b | 2.871^a^ | 2.997^a^ | ns | 2.909^a^ | 2.959^a^ | ns | No. of Spikelets/ plant | 46.7^b^ | 92.8^a^ | *** | 76.7^a^ | 62.8^b^ | * |
| Carotenoids/ Total Chlorophylls | 0.392^a^ | 0.216^b^ | *** | 0.191^b^ | 0.417^a^ | *** | Main Spike Mass (g) | 3.5^b^ | 4.2^a^ | * | 4.7^a^ | 3.0^b^ | *** |
| Chlorophyll Stability Index % | 74^b^ | 78^a^ | * | 100^a^ | 52^b^ | *** | Grain Yield/ Main Spike (g) | 2.8^b^ | 3.3^a^ | * | 3.8^a^ | 2.4^b^ | *** |
| A (µmol m^-2^ sec^-1^) | 7.3^b^ | 11.3^a^ | *** | 13.2^a^ | 5.4^b^ | *** | 100 kernel Mass (g) | 4.7^b^ | 5.1^a^ | *** | 5.3^a^ | 4.5^b^ | *** |
| E (mmol m^-2^ sec^-1^) | 1.0^a^ | 1.0^a^ | ns | 1.3^a^ | 0.7^b^ | *** | Biological Yield/ Plant (g) | 10.5^b^ | 21.2^a^ | *** | 18.5^a^ | 13.2^b^ | *** |
| pWUE (µmol mmol^-1^) | 7.1^b^ | 11.6^a^ | ** | 10.0^a^ | 8.7^a^ | ns | Straw Yield/ Plant (g) | 5.1^b^ | 11.0^a^ | *** | 9.0^a^ | 7.1^b^ | *** |
| Ls | 0.04^b^ | 0.26^a^ | *** | 0.18^a^ | 0.13^b^ | *** | Economic Yield/ plant (g) | 5.3^b^ | 10.2^a^ | *** | 9.5^a^ | 6.1^b^ | *** |
| gs (mmol m^-2^ sec^-1^) | 131^a^ | 95^b^ | *** | 164^a^ | 62^b^ | *** | Crop yield/ Plant (g) | 6.5^b^ | 12.6^a^ | *** | 11.6^a^ | 7.5^b^ | *** |
| gm (µmol mol^-1^) | 0.021^b^ | 0.043^a^ | *** | 0.047^a^ | 0.018^b^ | *** | Evapotranspiration Eff. | 0.9^b^ | 2.0^a^ | *** | 1.4^a^ | 1.5^a^ | ns |
| Ci (µmol mol^-1^ | 345^a^ | 266^b^ | *** | 297^b^ | 314^a^ | *** | WUE for Grain (g l^-1^) | 0.9^b^ | 1.9^a^ | *** | 1.5^a^ | 1.3^b^ | *** |
| Ci/ gs (µmol mmol^-1^ m^2^ sec) | 3.4^a^ | 3.5^a^ | ns | 1.8^b^ | 5.0^a^ | *** | WUE for Biomass (g l^-1^) | 1.9^b^ | 3.9^a^ | *** | 3.0^a^ | 2.8^a^ | ns |
| TSS (mg g^-1^) | 6.99^b^ | 8.10^a^ | *** | 6.9^b^ | 8.2^a^ | *** | grain TC (mg g^-1^ d wt) | 718.3^b^ | 779.8^a^ | *** | 785.1^a^ | 713.1^b^ | *** |
| ---------------------------- | ----- | ----- | ----- | ----- | ----- | ----- | grain TP (mg g^-1^ d wt) | 95.6^b^ | 125.1^a^ | *** | 102.1^b^ | 118.6^a^ | *** |

The data were represented as mean values, different superscript alphabetical letters refer to significant variation with the least significant difference (LSD) at *p* ≤ 0.05. The low, moderate, and high degrees of significance are indicated by *, **, and ***, while the non-significant difference is abbreviated as ns. Abbreviations: Dist. H_2_O (Distilled water), SPAE (*Spirulina platensis* aqueous extract), NI (normal irrigation), D (drought), A (photosynthesis rate), E (transpiration rate), pWUE (photosynthetic water use efficiency), Ls (stomatal limitation), gs (stomatal conductance), gm (mesophyll conductance), Ci (intercellular CO₂ concentration), TSS (total soluble sugars), TC (total carbohydrate content), Eff. (efficiency), and WUE (water use efficiency).

Supplementary Table 3 Interaction effect of grain priming and watering level on 49 morpho-physiological, and biochemical parameters of Shandawel 1 cultivar.

| parameters | Grain priming  ×  Watering Level | parameters | Grain priming  ×  Watering Level |
| --- | --- | --- | --- |
| Leaf Fresh mass (g) | * | Trehalose (mg g^-1^) | *** |
| Leaf Dry Mass (g) | ns | Polysaccharides (mg g^-1^) | *** |
| Succulence Degree (mg cm^-2^) | ns | Leaf TC (mg g^-1^) | *** |
| Sclerophylly Degree (mg cm^-2^) | *** | Plant Height (cm) | ns |
| Leaf Water Content (g g^-1^) | *** | Shoot Length (cm) | ns |
| Succulence Quotient (mg mg^-1^) | ** | Spike Length (cm) | ns |
| Leaf area (cm^2^) | *** | Peduncle Length (cm) | *** |
| Leaf Specific Area (cm^2^ g^-1^) | *** | No. of Tillers/ Plant | ns |
| Chlorophyll-a (mg g^-1^) | *** | No. of Grains/ Main spike | ns |
| Chlorophyll-b (mg g^-1^) | *** | No. of Grains/ Plant | ns |
| Carotenoids (mg g^-1^) | ns | No. of Spikes/ Plant | ns |
| Total Chlorophylls (mg g^-1^) | *** | No. of Spikelets/ Main spike | ns |
| Chlorophyll-a/ b | * | No. of Spikelets/ plant | ** |
| Carotenoids/ Total Chlorophylls | ** | Main Spike Mass (g) | ns |
| Chlorophyll Stability Index % | ns | Grain Yield/ Main Spike (g) | ns |
| A (µmol m^-2^ sec^-1^) | ** | 100 kernel Mass (g) | ns |
| E (mmol m^-2^ sec^-1^) | ns | Biological Yield/ Plant (g) | * |
| pWUE (µmol mmol^-1^) | ns | Straw Yield/ Plant (g) | ns |
| Ls | ns | Economic Yield/ plant (g) | ns |
| gs (mmol m^-2^ sec^-1^) | ns | Crop yield/ Plant (g) | * |
| gm (µmol mol^-1^) | ** | Evapotranspiration Eff. | ns |
| Ci (µmol mol^-1^ | ns | WUE for Grain (g l^-1^) | ns |
| Ci/ gs (µmol mmol^-1^ m^2^ sec) | * | WUE for Biomass (g l^-1^) | ns |
| TSS (mg g^-1^) | * | grain TC (mg g^-1^ d wt) | *** |
| ---------------------------- | ----- | grain TP (mg g^-1^ d wt) | ns |

The low, moderate, and high degrees of significance are indicated by *, **, and ***, while the non-significant difference is abbreviated as ns. Abbreviations: A (photosynthesis rate), E (transpiration rate), pWUE (photosynthetic water use efficiency), Ls (stomatal limitation), gs (stomatal conductance), gm (mesophyll conductance), Ci (intercellular CO₂ concentration), TSS (total soluble sugars), TC (total carbohydrate content), Eff. (efficiency), and WUE (water use efficiency).

Supplementary Table 4 Interaction effect of grain priming and watering level on 49 morpho-physiological, and biochemical parameters of Sakha 95 cultivar.

| parameters | Grain priming  ×  Watering Level | parameters | Grain priming  ×  Watering Level |
| --- | --- | --- | --- |
| Leaf Fresh mass (g) | *** | Trehalose (mg g^-1^) | *** |
| Leaf Dry Mass (g) | *** | Polysaccharides (mg g^-1^) | ** |
| Succulence Degree (mg cm^-2^) | ns | Leaf TC (mg g^-1^) | *** |
| Sclerophylly Degree (mg cm^-2^) | ns | Plant Height (cm) | * |
| Leaf Water Content (g g^-1^) | ns | Shoot Length (cm) | * |
| Succulence Quotient (mg mg^-1^) | ns | Spike Length (cm) | ns |
| Leaf area (cm^2^) | * | Peduncle Length (cm) | ns |
| Leaf Specific Area (cm^2^ g^-1^) | ns | No. of Tillers/ Plant | ns |
| Chlorophyll-a (mg g^-1^) | ** | No. of Grains/ Main spike | ns |
| Chlorophyll-b (mg g^-1^) | *** | No. of Grains/ Plant | * |
| Carotenoids (mg g^-1^) | ns | No. of Spikes/ Plant | ns |
| Total Chlorophylls (mg g^-1^) | *** | No. of Spikelets/ Main spike | *** |
| Chlorophyll-a/ b | ns | No. of Spikelets/ plant | ns |
| Carotenoids/ Total Chlorophylls | ** | Main Spike Mass (g) | ns |
| Chlorophyll Stability Index % | * | Grain Yield/ Main Spike (g) | ns |
| A (µmol m^-2^ sec^-1^) | *** | 100 kernel Mass (g) | ns |
| E (mmol m^-2^ sec^-1^) | ns | Biological Yield/ Plant (g) | ns |
| pWUE (µmol mmol^-1^) | ns | Straw Yield/ Plant (g) | ns |
| Ls | ns | Economic Yield/ plant (g) | ns |
| gs (mmol m^-2^ sec^-1^) | *** | Crop yield/ Plant (g) | ns |
| gm (µmol mol^-1^) | *** | Evapotranspiration Eff. | ** |
| Ci (µmol mol^-1^ | ns | WUE for Grain (g l^-1^) | ** |
| Ci/ gs (µmol mmol^-1^ m^2^ sec) | ns | WUE for Biomass (g l^-1^) | ** |
| TSS (mg g^-1^) | ** | grain TC (mg g^-1^ d wt) | *** |
| ---------------------------- | ----- | grain TP (mg g^-1^ d wt) | *** |

The low, moderate, and high degrees of significance are indicated by *, **, and ***, while the non-significant difference is abbreviated as ns. Abbreviations: A (photosynthesis rate), E (transpiration rate), pWUE (photosynthetic water use efficiency), Ls (stomatal limitation), gs (stomatal conductance), gm (mesophyll conductance), Ci (intercellular CO₂ concentration), TSS (total soluble sugars), TC (total carbohydrate content), Eff. (efficiency), and WUE (water use efficiency).
